# Supplementary material for: Post-pleistocene colonisation rather than the contemporary environment has most influenced the current population structure of Scottish Atlantic salmon (Salmo salar)
Source: PLoS One. 2025 Oct 1;20(10):e0333164. doi: 10.1371/journal.pone.0333164 (PMC12488018; doi:10.1371/journal.pone.0333164)
Supplement: S1 Table — (DOCX) [file pone.0333164.s001.docx]

Table.S1: Posterior estimates of regression parameters for the most probable models at each spatial scale.

| *All Sites* | | | | |
| --- | --- | --- | --- | --- |
| *Regression Coefficient* | *Factor* | *Mean* | *Mode* | *95% HPDI* |
| $\alpha_{0}$ | Constant | -4.14 | -3.89 | [-4.38; -3.91] |
| $\alpha_{5}$ | Time since deglaciation | 0.441 | 0.518 | [0.192; 0.652] |
| $\sigma^{2}$ | - | 0.58 | 0.522 | [0.333; 0.857] |
| *East Coast* | | | | |
| *Regression Coefficient* | *Factor* | *Mean* | *Mode* | *95% HPDI* |
| $\alpha_{0}$ | Constant | -4.42 | -4.42 | [-4.78; -4.02] |
| $\alpha_{5}$ | Time since deglaciation | 0.627 | 0.592 | [0.252; 1.01] |
| $\sigma^{2}$ | - | 0.658 | 0.54 | [0.272; 1.17] |
| *West Coast* | | | | |
| *Regression Coefficient* | *Factor* | *Mean* | *Mode* | *95% HPDI* |
| $\alpha_{0}$ | Constant | -3.88 | -3.86 | [-4.21; -3.55] |
| $\sigma^{2}$ | - | -0.692 | 0.632 | [0.335; 1.11] |
