# Supplementary material for: Post-pleistocene colonisation rather than the contemporary environment has most influenced the current population structure of Scottish Atlantic salmon (Salmo salar)
Source: PLoS One. 2025 Oct 1;20(10):e0333164. doi: 10.1371/journal.pone.0333164 (PMC12488018; doi:10.1371/journal.pone.0333164)
Supplement: Attachment — Submitted filename: PONE-D-24-37086_reviewer A.pdf [file pone.0333164.s003.pdf]

PLOS ONE

Post-Pleistocene Colonisation Rather than the Contemporary Environment has Most Influenced the Current Population Structure of Scottish Atlantic Salmon (Salmo salar)

--Manuscript Draft--

**Manuscript Number:**	PONE-D-24-37086
**Article Type:**	Research Article
**Full Title:**	Post-Pleistocene Colonisation Rather than the Contemporary Environment has Most Influenced the Current Population Structure of Scottish Atlantic Salmon (Salmo salar)
**Short Title:**	Colonisation of rivers by salmon
**Corresponding Author:**	Finn Cowell<br>University of St Andrews School of Biology<br>St Andrews, Fife UNITED KINGDOM OF GREAT BRITAIN AND NORTHERN IRELAND
**Keywords:**	Anadromous fish; Atlantic Salmon; Salmo salar; colonisation; genetic differentiation; contemporary factors
**Abstract:**	Genetic structuring in populations is the result of both historical and contemporary environmental factors driving genetic drift, natural selection and gene flow, as well as purely genetic factors, such as mutation and recombination. In Atlantic salmon (Salmo salar), re-colonisation of rivers after the last Ice Age was shown to be an important factor in shaping contemporary population structure, though the observed structure was more complex than was predicted through founder effects. Thus, other, perhaps more contemporary factors may also play a role. Here, we investigated the influence of both historical and contemporary factors on variation in population differentiation and diversity, based on microsatellite data (33 loci) collected from 48 Scottish Atlantic Salmon populations. The results confirmed the important effect of time since deglaciation but also uncovered the effect of waterbody modification, which differed between rivers draining on the east and west coasts of Scotland. Distance to the sea also had a non-negligible effect, while the other considered factors did not have a significant effect.
**Order of Authors:**	Finn Cowell<br>Oscar Eduardo Gaggiotti<br>Eef Cauwelier
**Additional Information:**	
**Question**	**Response**
**Financial Disclosure**<br><br>Enter a financial disclosure statement that describes the sources of funding for the work included in this submission. Review the submission guidelines for detailed requirements. View published research articles from *PLOS ONE* for specific examples.<br><br>This statement is required for submission and **will appear in the published article** if the submission is accepted. Please make sure it is accurate.	The author(s) received no specific funding for this work.

Powered by Editorial Manager® and ProduXion Manager® from Aries Systems Corporation

**Funded studies**<br>Enter a statement with the following details:<br>• Initials of the authors who received each award<br>• Grant numbers awarded to each author<br>• The full name of each funder<br>• URL of each funder website<br>• Did the sponsors or funders play any role in the study design, data collection and analysis, decision to publish, or preparation of the manuscript?<br><br>Did you receive funding for this work?	
**Competing Interests**<br><br>Use the instructions below to enter a competing interest statement for this submission. On behalf of all authors, disclose any competing interests that could be perceived to bias this work—acknowledging all financial support and any other relevant financial or non-financial competing interests.<br><br>This statement is required for submission and **will appear in the published article** if the submission is accepted. Please make sure it is accurate and that any funding sources listed in your Funding Information later in the submission form are also declared in your Financial Disclosure statement.<br><br>View published research articles from *PLOS ONE* for specific examples.	The authors have declared that no competing interests exist.

**NO authors have competing interests**<br><br>Enter: *The authors have declared that no competing interests exist.*<br><br>**Authors with competing interests**<br><br>Enter competing interest details beginning with this statement:<br><br>*I have read the journal's policy and the authors of this manuscript have the following competing interests: [insert competing interests here]*<br><br>* typeset	
**Ethics Statement**<br><br>Enter an ethics statement for this submission. This statement is required if the study involved:<br><br>• Human participants<br>• Human specimens or tissue<br>• Vertebrate animals or cephalopods<br>• Vertebrate embryos or tissues<br>• Field research<br><br>Write "N/A" if the submission does not require an ethics statement.<br><br>General guidance is provided below. Consult the submission guidelines for detailed instructions. **Make sure that all information entered here is included in the Methods section of the manuscript.**	The care and use of experimental animals complied with Scottish Government animal welfare laws, guidelines and policies as approved by Marine Scotland Science AWERB under Home Office project licence PP3525229.

Powered by Editorial Manager® and ProduXion Manager® from Aries Systems Corporation

**Format for specific study types**<br><br>**Human Subject Research (involving human participants and/or tissue)**<br>• Give the name of the institutional review board or ethics committee that approved the study<br>• Include the approval number and/or a statement indicating approval of this research<br>• Indicate the form of consent obtained (written/oral) or the reason that consent was not obtained (e.g. the data were analyzed anonymously)<br><br>**Animal Research (involving vertebrate animals, embryos or tissues)**<br>• Provide the name of the Institutional Animal Care and Use Committee (IACUC) or other relevant ethics board that reviewed the study protocol, and indicate whether they approved this research or granted a formal waiver of ethical approval<br>• Include an approval number if one was obtained<br>• If the study involved *non-human primates*, add *additional details* about animal welfare and steps taken to ameliorate suffering<br>• If anesthesia, euthanasia, or any kind of animal sacrifice is part of the study, include briefly which substances and/or methods were applied<br><br>**Field Research**<br><br>Include the following details if this study involves the collection of plant, animal, or other materials from a natural setting:<br>• Field permit number<br>• Name of the institution or relevant body that granted permission	
**Data Availability**<br><br>Authors are required to make all data underlying the findings described fully available, without restriction, and from the time of publication. PLOS allows rare exceptions to address legal and ethical concerns. See the PLOS Data Policy and FAQ for detailed information.	Yes - all data are fully available without restriction

A Data Availability Statement describing where the data can be found is required at submission. Your answers to this question constitute the Data Availability Statement and **will be published in the article**, if accepted.<br><br>**Important:** Stating 'data available on request from the author' is not sufficient. If your data are only available upon request, select 'No' for the first question and explain your exceptional situation in the text box.<br><br>Do the authors confirm that all data underlying the findings described in their manuscript are fully available without restriction?	
**Describe where the data may be found in full sentences. If you are copying our sample text, replace any instances of XXX with the appropriate details.**<br><br>• If the data are **held or will be held in a public repository**, include URLs, accession numbers or DOIs. If this information will only be available after acceptance, indicate this by ticking the box below. For example: *All XXX files are available from the XXX database (accession number(s) XXX, XXX.).*<br>• If the data are all contained **within the manuscript and/or Supporting Information files**, enter the following: *All relevant data are within the manuscript and its Supporting Information files.*<br>• If neither of these applies but you are able to provide **details of access elsewhere**, with or without limitations, please do so. For example:<br><br>*Data cannot be shared publicly because of [XXX]. Data are available from the XXX Institutional Data Access / Ethics Committee (contact via XXX) for researchers who meet the criteria for access to confidential data.*<br><br>*The data underlying the results presented in the study are available from (include the name of the third party*	All relevant data are within the manuscript and its Supporting Information files.

and contact information or URL).<br>• This text is appropriate if the data are owned by a third party and authors do not have permission to share the data.<br><br>* typeset	
Additional data availability information:	

Powered by Editorial Manager® and ProduXion Manager® from Aries Systems Corporation

Manuscript

Click here to access/download;Manuscript;PLOS submission with vancouver style.docx

Post-Pleistocene Colonisation Rather than the Contemporary Environment has Most Influenced the Current Population Structure of Scottish Atlantic Salmon (*Salmo salar*)

Author list: Finn Cowell[1*], Oscar E. Gaggiotti [1] and Eef Cauwelier[2]

[1] Centre for Biological Diversity, School of Biology, University of St Andrews, St Andrews, Fife, Scotland, United Kingdom

[2] Marine Directorate, Freshwater Fisheries Laboratory, Faskally, Pitlochry, Perthshire, Scotland, United Kingdom

Corresponding author: Finn Cowell (ffmc@st-andrews.ac.uk)

Abstract

Genetic structuring in populations is the result of both historical and contemporary environmental factors driving genetic drift, natural selection and gene flow, as well as purely genetic factors, such as mutation and recombination. In Atlantic salmon (*Salmo salar*), re-colonisation of rivers after the last Ice Age was shown to be an important factor in shaping contemporary population structure, though the observed structure was more complex than was predicted through founder effects. Thus, other, perhaps more contemporary factors may also play a role. Here, we investigated the influence of both historical and contemporary factors on variation in population differentiation and diversity, based on microsatellite data (33 loci) collected from 48 Scottish Atlantic Salmon populations. The results confirmed the important effect of time since deglaciation but also uncovered the effect of waterbody modification, which differed between rivers draining on the east and west coasts of Scotland. Distance to the sea also had a non-negligible effect, while the other considered factors did not have a significant effect.

Keywords: Anadromous fish, Atlantic Salmon, *Salmo salar*, colonisation, genetic differentiation, contemporary factors

Introduction

Genetic relationships between populations are shaped by processes that either tend to increase differentiation, such as natural selection, genetic drift, founder effects and mutation or decrease it, such as gene flow through migration. In populations of Atlantic salmon (*Salmo salar*), the impact of these forces can be marked, as the homing behaviour they exhibit can lead to significant genetic structuring, with individuals tending to return to their natal rivers to spawn [1]. This may result in relatively low levels of gene flow and would allow mutations

to accumulate, genetic drift to randomly fix alleles and natural selection to lead to adaptation to the unique local environment [2,3,4]

Anadromous salmonids have colonised and re-colonised rivers following the respective retreat and expansion of ice sheets [5] Indeed, the influence of ice sheet movement on genetic structure in Atlantic salmon populations has been reported in the Baltic Sea [6], Iceland [7] the British Isles [8,9] and, most recently, Norway and the areas of Russia surrounding the Barents and White seas [10]. This observed population genetic structure has been attributed to strong founder effects upon colonisation of newly ice-free rivers [11].

Initial colonisation and associated establishment of populations will have been further influenced by factors related to deglaciation, such as isostatic rebound, which would have affected river accessibility [12,13] and population connectivity. Isostatic rebound resulted in relative sea levels decreasing [13] resulting in some of the lower stretches of contemporary rivers being saltwater areas that were not colonisable by Atlantic salmon until long after the ice sheets retreated. Concurrently, higher reaches of rivers, with lower carrying capacity, might have become available as the ice sheet retreated, expanding the species' range upstream. It is therefore uncertain how the gradual expansion within a river affected the relationship between distance to sea and differentiation, as well as population connectivity.

Despite Cauwelier *et al.* [8] identifying the movement of ice sheets as having an important influence on genetic structuring on Scottish *S. salar* populations, ecological niche modelling could not explain all the differences between populations. Contemporary factors, such as climate change, waterbody modification, etc. are, therefore, of interest, as there have been

many anthropogenic impacts on watercourses since the time of deglaciation and it is possible that these have resulted in genetic changes in *S. salar* populations.

One such anthropogenic impact is that of post-industrial revolution climate change, which may be among the drivers of the rising river temperatures documented in Scotland during the 20th and 21st century [14]. In some cases, local adaptation to thermal regimes has been shown to result in greater genetic diversity within and differentiation between *S. salar* populations [15]. However, the reported effect of temperature may be indirect rather than causal, as it affects other elements of the ecosystem, such as bacterial diversity [16].

Next to water temperature, water quality is also an important factor influencing the survival of anadromous fish and associated population diversity and differentiation. Despite the lack of research detailing the specific impact of water quality on genetic structuring, poor water quality has been shown to restrict distribution [17] and reduce abundance [18] in anadromous fishes.

Further direct and indirect effects of anthropogenic activity, such as modifications to waterways (e.g. dams – [19,20,21] and population interbreeding due to stocking [22] and/or farm escapees associated with aquaculture [23,24,25] also impact genetic structuring.

Lastly, efforts to protect salmon populations are expected to also have an impact on the species' diversity and structuring, whether through stocking and/or habitat conservation/restoration. Attempts to protect *S. salar* in Scotland, such as the SAC (Special Area of Conservation) designation, focus primarily on habitat conservation and sound land

management. The impact of these designations on salmon populations is, however, poorly understood.

All the above factors, both historical and contemporary, have the potential to alter levels of gene flow and genetic drift. These evolutionary forces can, in turn, influence the genetic composition of extant populations. However, the possible relationship between genetic differentiation and environmental factors, both historical and contemporary has not been considered concurrently. Here, we investigated the effect of both historical and contemporary factors on Scottish Atlantic salmon population structure. Data for 48 *S. salar* populations from across Scotland were used to calculate measures of genetic differentiation and diversity. The impacts of the time since deglaciation, distance to the sea, population connectivity, temperature, water quality, waterbody modification, and environmental protections were assessed through general linear modelling using both a frequentist and a Bayesian approach.

Methods

Sample Collection and Screening

Due to computational demands required for the Bayesian approach, the full dataset described in Cauwelier *et al.* [8] was not used. As such, sites were randomly selected to encompass the genetic groups identified by Cauwelier *et al.* [8] and this dataset was extended by screening for an additional sixteen microsatellites, in order to increase genome coverage.

Between 2003 and 2010, 48 sites across 41 Scottish rivers were electrofished for juvenile salmon (Figure 1). Fin clips were taken from anaesthetized fish, the fish released and clips stored in 99% ethanol. DNA was extracted using the method described by Knox *et al.* [26]

and initially amplified for three multiplexes encompassing fourteen microsatellites ([27]- excluding *SsaD486*) plus *SsaD48*, *SsaD71* [28] and *SP1608* [29] Full details on PCR conditions and fragment analysis are described in Cauwelier *et al.* [8]. Screening for an additional sixteen microsatellites (*EST107*, *EST19,EST28*, *EST68*, *MHC1*, *MHC2, Sleel53, Sleen82*, *Ssa405*, *Ssa407*, *Ssa412*, *Ssa98*, *Ssleer15*, *SsOSL25*, *Ssosl85* and *SsSP2215*) was carried out at the Norwegian Institute for Marine Research, using the protocol described in Harvey *et al.* [30]. The resulting dataset consisted of 1,044 fish genotyped at 33 microsatellite loci.

Figure 1: Map of Scotland (1: 2,000,000) and its location within Europe (inset), depicting the electrofished sites [31] Numbers relate to site numbers detailed in Table S1.

<u>Genetic Analysis</u>

Tests for deviation from Hardy-Weinberg (HW) equilibrium were conducted using the Markov chain method in the Genepop package [32] in R version 4.0.3 [33]. This was run with 2,000 iterations of 500 batches with a dememorization length of 10,000. The same package was used to test for linkage disequilibrium (LD), with 1,000 iterations over 100 batches and a dememorization of 10,000. Handling multiple tests to avoid false positives was done by following Waples [34], using the cumulative binomial distribution to identify if the number of positive tests significantly exceeded those expected due to chance alone.

Rarefied allelic richness was calculated using hierfstat [35]. Population-specific differentiation was based on F_{st} values, which were calculated using GESTE [36]. GESTE is

a Bayesian programme that estimates population-specific F_{st} values on the basis of how divergent the population is from all the other sites.

Environmental Data

Environmental data were synthesised from several sources. Data on water quality and protection status were obtained from NatureScot [37]. Data on the water quality in rivers used the Water Framework Directive classification of a water body as either High, Good, Moderate, Poor or Bad. It also designates if a water body is heavily modified. This same data website provided information from the Scottish Environmental Protection Agency (SEPA) on the protection status of rivers and surrounding areas. The designated protection for each site was recorded and, if the reason for this designation included the need to protect salmon populations, then this information was also recorded. The qualitative descriptors of habitat protection were coded as three binary dummy variables. The first variable showed if salmon were specially protected or not in that section of river, the second detailed if the section of river was protected for a reason unrelated to salmon or not, and the third detailed if the river, as well as a broader area surrounding the river, was protected or not.

Water temperature data came from the Scotland River Temperature Monitoring Network, through the Marine Scotland Website [38]. These are modelled data that have been inferred from strategic monitoring of temperature at specific sites throughout Scotland and the consideration of influential landscape features [39]. The figures used were the highest maximum temperature over the last 20 years. As well as this, the time since deglaciation was determined from maps of the deglaciation process made by Cauwelier *et al.* [8].

Distance to the sea and elevation data (OS terrain 50 layer) were calculated using ArcMap 10.6 [40]. Pairwise distances were calculated by measuring the shortest swimmable distance from the mouth of the rivers using Free Map Tools [41]. This was then added to the distance to the sea measures of the two rivers. This pairwise distance matrix was then converted into a population-specific measure of connectivity, which was calculated as the mean of all pairwise distances between a population and all other populations.

In order to limit issues arising from multicollinearity, a correlation matrix was calculated between all the environmental variables. One variable of each pair was removed in cases of a correlation coefficient greater than 0.8. Furthermore, variables with a variance inflation factor (VIF) greater than 5 were also removed.

Modelling

Diversity data were analysed by using General Linear Models (GLMs), with all environmental variables initially included. A procedure of backwards elimination was employed, using a p-to-remove criterion, until only a single independent variable was included in the remaining model. The best model was then chosen as the one with the lowest Akaike's Information Criterion (AIC). All GLMs assumed a normal distribution, as this best fitted the model assumptions. As many of the variables were categorical, the *drop1* function in R was used, as this added and removed each term of the model in turn, allowing the effect of that factor on the R^2 to be realised and the significance to be determined.

GESTE was used to investigate the association between population-specific F_{st} and environmental factors following the procedure described in [42]. This involved using ten pilot runs of 1,000 iterations, a burn in period of 5,000,000 iterations and a thinning interval

of 50. An initial exploratory run with all factors (full model) was used to identify the five factors with the highest marginal posterior probability across all models, which were then used in a second run. The five models with the highest posterior probabilities were reported.

For both population-specific differentiation and diversity analysis, different spatial scales were analysed. Initially, all rivers were included, which encompassed seven genetic regions [8]. A hierarchical structure was then considered, whereby data were split into rivers draining on the east coast and west coast and analysed separately. Carrying out separate analyses for the two geographic regions was done in order to avoid confounding effects by factors that could not be accounted for by statistical analyses, given that the anthropogenic effects were likely to differ between the two coasts. In particular, protection of waterbody for a reason other than salmon, as well as protection of the surrounding land, only applied to the west coast. Moreover, the geomorphology of east and west coasts has long been acknowledged to differ [43]. The three sites on the north coast of Scotland were categorised as being in the west coast group, as they form part of a genetic grouping extending west [8].

Results

Genetic Diversity and Differentiation

HW deviations and positive tests for LD were not significantly greater than the number expected due to effects of chance alone. Mean rarefied allelic richness equalled 5.47 (SD = 0.25). Population-specific F_{st} generated from GESTE, when all sites were considered, ranged from 0.002 in the River Don to 0.084 in the River Leven with a mean F_{st} of 0.021 (SD = 0.019). Full diversity and differentiation results can be found in Table S1.

General linear modelling

Variation in rarefied allelic richness at all sites was best explained by a model considering time since deglaciation and distance to the sea, the former being statistically significant (Figure 2 and Table 1). In the case of the east coast, waterbody modification was also included in the best model, which was more significant than the time since deglaciation and the former also had a greater sum of squares (0.23) than the latter (0.09). Genetic diversity on the west coast meanwhile was best predicted by the sole factor of time since deglaciation. The R^2 for the east coast model was more than twice as high when compared to the all-sites or west coast models.

Table.1: Summary of results from the best GLMs for each coast, explaining rarefied allelic richness patterns using backwards elimination.

			All Sites		
Predictors	*Term df*	*Residual df*	*F-value*	*Adjusted R-squared*	*P-value*
Distance to Sea	1	46	3.02		0.089
Time since Deglaciation	1	46	19.3	0.29	< **0.001**
			East Coast		
Predictors	*Term df*	*Residual df*	*F-values*	*Adjusted R-squared*	*P-value*
Waterbody Modification	1		11.6		**0.003**
Distance to the Sea	1	18	2.83	0.603	0.11
Time since Deglaciation	1		4.53		**0.047**
			West Coast		
Predictors	*Term df*	*Residual df*	*F-value*	*Adjusted R-squared*	*P-value*
Time Since deglaciation	1	24	9.08	0.244	**0.006**

Figure.2: The relationship of rarefied allelic richness and the timing since deglaciation (before present - BP) in salmon sampled from Scottish rivers adjusted for all the variables considered by the best model (as described in table 1). Full line represents the mean, whilst dashed lines represent one standard error from the mean.

When all sites were included, the marginal posterior probabilities of each factor, when GESTE considered all nine (Table 2), was greatest for time since deglaciation. This was also true when considering the east and west coasts separately. However, additionally, on the east coast, waterbody modification and distance to the sea also had non-negligible posterior probabilities, and the former had a higher posterior probability than the latter.

Table.2: The sum of marginal posterior probabilities that include a given factor, produced by GESTE when all nine variables were included.

Factors	*Sum of Marginal Posterior Probabilities*		
	All Sites	*East Coast*	*West Coast*
Time since deglaciation	0.596	0.524	0.533
Distance to the sea	0.194	0.144	0.063
Water quality	0.053	0.044	0.069
Waterbody modification	0.04	0.162	0.063
Connectivity	0.029	0.035	0.057
Protection of surroundings	0.023	N/A	0.061
Protection of salmon	0.022	0.044	0.064
Protection of the waterbody	0.022	N/A	0.045
Temperature	0.02	0.032	0.045

Subsequent analysis of the effect of the five best environmental variables on population-specific F_{st} across all sites also revealed the predominant influence of time since deglaciation (Table 3) but distance to the sea was also found to be an influential variable. Population F_{st}

decreased with the time since deglaciation and with distance from the sea (Figure 3). This was also the case when the east coast was considered separately where, additionally, waterbody modification was important (Figure 3, Table 3).

Table.3: Posterior probabilities of the five most probable models for the site-specific GESTE analysis when considering five factors, at three different spatial scales

	All Sites	
Model	*Factors Included*	*Posterior Probability*
17	Time since deglaciation	0.549
21	Time since deglaciation and distance to the sea	0.296
18	Time since deglaciation and water quality	0.048
19	Time since deglaciation and waterbody modification	0.032
22	Time since deglaciation, distance to the sea and water quality	0.02
	East Coast	
	Factors Included	*Posterior Probability*
17e	Time since deglaciation	0.405
25e	Time since deglaciation and distance to the sea	0.171
19e	Time since deglaciation and waterbody modification	0.155
21e	Time since deglaciation and protection of salmon	0.063
3e	Waterbody modification	0.047
	West Coast	
	Factors Included	*Posterior Probability*
1w	Null	0.43
17w	Time since deglaciation	0.318

9w	Distance to the sea	0.041
3w	Waterbody modification	0.038
2w	Water Quality	0.031

Figure.3: Results from all the GESTE models with a non-negligible posterior probability with means and one standard error from the mean represented. Model numbers relate to the numbers in Table 3.

Indeed, unmodified waterbodies exhibited much lower genetic differentiation than those that have been modified. On the other hand, the results from the west coast dataset showed that the null model was best. The marginal posterior probability on the west coast for waterbody modification, distance to the sea and time since deglaciation were 0.099, 0.1 and 0.598, respectively. In this same order, the east coast results were 0.172, 0.152 and 0.567.

Discussion

The aim of this study was to explore the major historical and contemporary factors contributing to genetic differentiation and diversity in *S. salar*. Overall, the largest factor associated with the observed diversity/differentiation was timing since deglaciation, which was also reported in Cauwelier et al. [8]. However, contemporary factors also seemed to be

playing a role, particularly when considering rivers draining in the east coast of Scotland, where waterbody modification had a non-negligible effect on both genetic diversity and differentiation. Additionally, an effect of distance to sea was also detected but its effect was very small and appeared to be driven by outlier sites.

Post-Pleistocene Colonisation

Previous studies have shown that the founder effects associated with recolonisation of newly ice-free rivers [6,7,8,10] are still the predominant determinant of contemporary genetic structuring in Atlantic salmon populations. These results, showing low genetic diversity and high differentiation in the most recently deglaciated rivers, is akin to the stepping-stone model [44] and central-marginal hypothesis [45]. This result is likely to be independent of the type of molecular markers used, as SNP based analysis [46] resulted in very similar groupings to when microsatellites were used [8].

Distance to the Sea

Although distance to the sea was not found to have a significant effect on genetic diversity, it did have a non-negligible posterior probability (all sites and east coast) in the GESTE analysis. However, the slopes (figure 3 and table S2) were very close to zero, which would suggest that the distance from the sea was not biologically meaningful. In both cases, the influence of the distance to the sea was accompanied by the time since deglaciation, which was unsurprising, given that colonisation might majorly have been in an upstream direction, as the ice sheet was receding. However, the results suggest that genetic differentiation decreased with increasing distance to the sea; the opposite of what previous studies have found [15, 47].

Indeed, according to population genetics theory and previous empirical studies, a positive relationship between differentiation and distance to the sea can be expected. This is because colonisation upstream will, historically, have been impeded by the ice sheets. However, the river itself would also have experienced elongation after deglaciation, due to the isostatic rebound [12,13]. As such, subsequent colonisation would have occurred both upstream and downstream but patterns might have differed depending on the carrying capacity of the newly available habitat, which, most likely, would have been larger downstream than upstream. This, in turn, may have resulted in higher gene flow and reduced impact of founder effects and drift in downstream populations compared to upstream colonisation. These processes would then result in a positive relationship between differentiation and distance to sea, which would, in turn, be related to timing of deglaciation.

The unexpected negative relationship between differentiation and distance to sea could be due to outlier sites. These outlier sites were characterised by high population-specific F_{st} close to the mouth of the river or low F_{st} at large distances from the sea. Coincidentally, the outlier sites were among the first (low F_{st}) or the most recent (high F_{st}) to become ice free. Indeed, four of the five sites (Awe, Howmore, Leven and Strontian) showing high F_{st} close by the mouth of the river were deglaciated recently. Additionally, these rivers are all small west coast rivers, which are likely to harbour smaller populations. Small rivers with low carrying capacities have been shown to have highly differentiated *S. salar* populations, due to stronger genetic drift in these populations [48,49]. Smaller rivers could also be the reason why the impact of distance to the sea was negligible on the west coast.

Conversely, four sites on the rivers Spey (Dulnain, Feshie), Don and Tweed, were characterised by low F_{st} values and large distances from the sea. These are, unsurprisingly, all part of large, east coast drainage systems. Previous tagging of fish within the drainage systems of north-east Scotland (including the Spey and Don) has identified high straying rates which are largely responsible for the low levels of genetic differentiation within and between these highly productive rivers [50].

Overall, the fact that such outliers exist suggests that the effect of the distance to the sea is overridden as an influence on genetic differentiation by the effect of time since deglaciation. When the outliers were removed, the relationship between differentiation and distance to the sea became positive. Although not statistically significant ($r = 0.237$, $p = 0.159$), this is more in line with the previous findings [15,47].

Waterbody Modification

The impact of waterbody modification on genetic diversity and differentiation was only observed in the east coast analysis. The fact that genetic diversity on the east coast is significantly reduced at sites that have been heavily modified suggests, unsurprisingly, that there have been impediments to gene flow as a result of these modifications. The non-negligible posterior probability of the time since deglaciation and waterbody modification GESTE model further shows how these two factors can interact to create the pattern of differentiation seen today. Sites that have been deglaciated recently have been the major contributor to genetic differentiation, but waterbody modifications have likely reduced gene flow between populations, increasing differentiation and reducing diversity further.

There were differences in the nature of the modifications between both coasts, with hydro schemes dominating the modifications along the east coast, whilst modifications for flood prevention and agriculture were more frequently found along the west coast. Indeed, hydro schemes were responsible for five of the six alterations on the east coast sites, whereas this is only true for four out of the seven modified sites on the west coast. The absence of an impact of waterbody modifications on diversity/differentiation along the west coast could suggest that these cases of flood prevention and irrigation have not significantly altered the genetic composition of these populations. Furthermore, the results provide another example that hydro schemes can have a marked impact on genetic structuring [19,20,21].

<u>Other Factors</u>

The other factors considered in the model, water quality, temperature and the various environmental protections, appeared to have had a negligible impact on genetic structuring. This would suggest that these particular anthropogenic activities have not been important drivers of genetic structuring in this species.

It should be noted that more detailed and varied data underlying some of the categorical variables considered here may have revealed patterns which this cruder analysis failed to detect. For example, water quality used in this study was a composite measure based on numerous indicators, such as direct measurements of chemicals and the presence of invertebrates. Therefore, water quality may not have been designated as poor when only a

single pollutant was found in high concentration and the other pollutants were at a low concentration. A better consideration would have been to collect data on levels of pollutants known to negatively affect salmon survival.

Contrasting results have been reported on the effect of temperature on genetic differentiation. Dionne *et al.* [15] showed that increases in genetic differentiation were associated with higher temperatures in some *S. salar* populations. In contrast, anadromous rainbow (steelhead) trout (*Oncorhynchus mykiss)* showed reduced differentiation with increased temperature [51]. This would suggest that more research is needed into the effect of temperature on population structuring in various species of Salmonids.

In general, although many environmental factors were considered in this study, these were often categorical and therefore encapsulated a lot of variability. Furthermore, there were other environmental variables left unincluded due to a paucity of data and/or the level of complexity, such as those relating to hydrology and geography. For example, populations in close proximity to one another but located across geological boundaries have been shown to be more differentiated than populations within them, which might be linked to accuracy of homing [52]. Further fine-tuning of the various categorical factors and incorporating hydrological and geological parameters should, in future studies, shed more light on their impact on contemporary diversity and structuring of *S. salar* populations.

<u>West coast</u>

Similar to the analysis of all sites and the east coast sites, the results from the west coast analysis showed an effect of timing since deglaciation on genetic diversity, with lower diversity found in the most recently deglaciated rivers. It is, however, notable that the R^2 more than doubles west coast sites are not included. It might be that the limited variability of the various factors considered played a role. For example, distance to sea varied from 0.113 to 58.5 km (mean = 14.9 and SD = 16.5) along the west coast, whilst this was between 2.75 and 121 km (mean = 42.2 and SD = 36.3) along the east coast. However, the low R^2, together with the finding that the null model was the best when looking at population-specific F_{st}, might also suggest that, despite including many factors in this study, some other, more prominent factors, were missed.

One factor that was not considered in the model was the presence of aquaculture. Indeed, fish farms are almost exclusively located on the west coast of Scotland. Given that Gilbey *et al.* [53], have detected introgression from farmed fish in wild west coast populations, it cannot be ruled out that aquaculture has had a significant impact on the populations in this study. Glover *et al.* [25] showed that, in Norway, the presence and growth of the aquaculture industry has led to an erosion of wild salmon population structure over time, with contemporary populations being less differentiated than their historical counterparts. However, the impact of aquaculture on wild populations varies across regions [23,53] and is very complex to measure and model [54,55]. As such, it would be very difficult to disentangle and robustly attribute the impact of aquaculture from the other local environmental characteristics of the rivers in question.

Conclusions

In summary, the results from this study have reinforced the findings of Cauwelier *et al.* [8] but puts the influence of post-Ice Age colonisation in the context of the other contemporary forces considered and differences in the type of anthropogenic perturbations between east and west river-drainage systems in Scotland. In this regard, the most important contemporary factor is waterbody modification affecting rivers draining in the east. However, other river characteristics and anthropogenic factors not considered in this study could also be important and these should be explored in more detail.

Acknowledgements: Thanks to Widar Wennevik for helpful comments and discussion. Thanks also to John Gilbey for assisting in the selection of the sample sites and to Synne Winterthun for her work on screening and QC-ing the samples for the additional microsatellites. This work was made possible due to the partial sample screening carried out under the Research Council of Norway project 280308 (SeaSalar).

References

1. Klemetsen A, Amundsen P, Dempson JB, Jonsson B, Jonsson N, O'Connel MF, et al. Atlantic salmon *Salmo salar* L., brown trout *Salmo trutta* L. and Arctic charr *Salvelinus alpinus* (L.): a review of aspects of their life histories. Ecology of Freshwater Fish. 2003;12:1-59.
2. Garcia de Leaniz C, Fleming IA, Einum S, Verspoor E, Jordan WC, Consuegra S, et al. A critical review of adaptive genetic variation in Atlantic salmon: implications for conservation. Biological Reviews. 2007;82: 173-211.

3. Landry C, Bernatchez L. Comparative analysis of population structure across environments and geographical scales at major histocompatibility complex and microsatellite loci in Atlantic salmon (*Salmo salar*). Molecular Ecology. 2001;10: 2525-2539.
4. Verspoor E, Beardmore JA, Consuegra S, Garcia de Leaniz C, Hindar K, Jordan W, et al. Population structure in the Atlantic salmon: insights from 40 years of research into genetic protein variation. Journal of Fish Biology. 2005;67: 3-54.
5. McDowall RM. Why are so many boreal freshwater fishes anadromous? Confronting 'conventional wisdom'. Fish and Fisheries. 2008;9: 208-213.
6. Säisä M, Koljonen M-L, Gross R, Nilsson J, Tahtinen J, Koskiniemi J, et al. Population genetic structure and postglacial colonization of Atlantic salmon (*Salmo salar*) in the Baltic Sea area based on microsatellite DNA variation. Canadian Journal of Fisheries and Aquatic Sciences. 2005; 62: 1887-1904.
7. Olafsson K, Pampoulie C, Hjorleifsdottir S, Gudjonsson S, Hreggvidsson GO, Present-day genetic structure of Atlantic salmon (*Salmo salar*) in Icelandic rivers and ice-cap retreat models. PLOS ONE. 2014;9: e86809
8. Cauwelier E, Verspoor E, Coulson MW, Armstrong A, Knox D, Stradmeyer L, et al. Ice sheets and genetics: insights into the phylogeography of Scottish Atlantic salmon, *Salmo salar* L.. Journal of Biogeography 2018;45: 51-63.
9. Finnegan AK, Griffiths AM, King RA, Machado-Schiaffino G, Porcher J-P, Garcia-Vazquez E, et al. Use of multiple markers demonstrates a cryptic western refugium and postglacial colonisation routes of Atlantic salmon (*Salmo salar* L.) in northwest Europe. Heredity. 2013;111: 34-43.

10. Wennevik V, Quintela M, Skaala Ø, Verspoor E, Prusov S, Glover KA, Population genetic analysis reveals a geographically limited transition zone between two genetically distinct Atlantic salmon lineages in Norway. Ecology and Evolution. 2019;9: 6901-6921.
11. Bernatchez L, Wilson CC, Comparative phylogeography of 22 nearctic and palearctic fishes. Molecular Ecology. 1998;7: 431-452.
12. Shennan I, Bradley S, Milne G, Brooks A, Bassett S, Hamilton S. Relative sea-level changes, glacial isostatic modelling and ice-sheet reconstructions from the British Isles since the last glacial maximum. Journal of Quaternary Science 2006;21: 585-599.
13. Shennan I, Hamilton S, Hillier C, Hunter A, Woodall R, Bradley S, et al . Relative sea-level observation in western Scotland since the last glacial maximum for testing models of glacial isostatic land movements and ice-sheet reconstructions. Journal of Quaternary Science. 2006;21: 601:613.
14. Pohle I, Helliwell R, Aube C, Gibbs S, Spencer M, Spezia L. Citizen science evidence from the past century shows that Scottish rivers are warming. Science of the Total Environment. 2019;659: 53-65.
15. Dionne M, Caron F, Dodson JJ, Bernatchez L. Landscape genetics and hierarchical genetic structure in Atlantic Salmon: the interaction of gene flow and local adaptation. Molecular Ecology. 2008;17: 2382-2396.
16. Dionne M, Miller KM, Dodson JJ, Caron F, Bernatchez L. Clinical variation in MHC diversity with temperature: evidence for the role of host- pathogen interaction on local adaptation in Atlantic salmon. Evolution. 2007;61: 2154- 2164.
17. Maes J Stevens M, Breine J. Poor water quality constrains the distribution and movements of twaite shad *Alosa fallax fallax* (Lacépède, 1803) in the watershed of river Scheldt. In Durfour S, Prévost E, Rochard E, Williot P. (Eds), Fish and Diadromy in

Europe (ecology, management, conservation). Developments in Hydrobiology, vol 200. 602: 155-159. Dordrecht: Springer; 2008.

18. Zdasiuk BJ, Chen CY, McCormick SD, Nislow KH, Singley JG, Kelly JT. Evaluating acid-aluminium stress in streams of the Northeastern U.S. at watershed, fish community and physiological scales. Ecological Indicators. 2002;144: 109480.
19. Deiner K, Garza JC, Coey R, Girman DJ. Population structure and genetic diversity of trout (*Oncorhynchus mykiss*) above and below natural and man-made barriers in the Russian River, California. Conservation Genetics. 2007;8: 437-454.
20. Samad-zada F, van Poorten BT, Harris S, Godbout L, Russello MA. Genome-wide analysis reveals demographic and life-history patterns associated with habitat modification in landlocked deep-spawning sockeye salmon (*Oncorhynchus nerka*). Ecology and Evolution. 2021; 11: 13186-13205.
21. Yamamoto S, Morita K, Koizumi I, Maekawa K. Genetic differentiation of white-spotted charr (*Salvelinus leucomaenis*) populations after habitat fragmentation: Spatial-temporal changes in gene frequencies. Conservation Genetics. 2004;5: 529-538.
22. Cross TF, McGinnity P, Coughlan J, Dillane E, Ferguson A, Koljonen M, et al. Stocking and Ranching. In Verspoor E, Stradmeyer L, Nielsen JL. (Eds), The Atlantic Salmon: Genetics, Conservation and Management. 325-356, Oxford: Blackwell Publishing; 2007.
23. Diserud OH, Fiske P, Karlsson S, Glover KA, Naesje T, Aronsen T, et al. Natural and anthropogenic drivers of escaped farmed salmon occurrence and introgression into wild Norwegian Atlantic salmon populations. ICES Journal of Marine Science. 2022;79: 1363-1379.
24. Ferguson A, Fleming IA, Hindar K, Skaala Ø, Mcginnity P, Cross T, et al. Farm Escapes. In Verspoor E, Stradmeyer L, Nielsen JL. (Eds), The Atlantic Salmon: Genetics, Conservation and Management. 357-398, Oxford: Blackwell Publishing: 2007.

25. Glover KA, Quintela M, Wennevik V, Besnier F, Sørvik AGE, Skaala Ø. Three decades of farmed escapees in the wild: A spatio-temporal analysis of Atlantic Salmon genetic structure throughout Norway. PLOS ONE. 2012;7: e43129.

26. Knox D, Lehmann K, Reddin DG, Verspoor E. Genotyping of archival Atlantic salmon scales from northern Quebec and West Greenland using novel PCR primers for degraded mtDNA. Journal of Fish Biology. 2002;60: 266-270.

27. Ellis JS, Gilbey J, Armstrong A, Balstad T, Cauwelier E, Cherbonnel C, et al. Microsatellite standardization and evaluation of genotyping error in a large multi-partner research programme for conservation of Atlantic salmon (*Salmo salar* L.). Genetica. 2011;139: 353-367.

28. King TL, Eackles MS, Letcher BH. Microsatellite DNA markers for the study of Atlantic salmon (Salmo salar) kinship, population structure, and mixed-fishery analyses. Molecular Ecology Notes. 2005;5: 130-132.

29. Paterson S, Piertney SB, Knox D, Gilbey J, Verspoor E. Characterization and PCR multiplexing of novel highly variable tetranucleotide Atlantic salmon (Salmo salar L.) microsatellites. Molecular Ecology Notes. 2004;4: 160-162.

30. Harvey, A. C. Quintela, M., Glover, K. A. Karlsen, Ø. Nilsen, R. Skaala, Ø. Sægrov, H. Kålås, H. Knutar, S. and Wennevik, V. Inferring Atlantic salmon post-smolt migration patterns using genetic assignment. Royal Society open science. 2019;6: 190426.

31. QGIS Development Team QGIS Geographic Information System. Open Source Geospatial Foundation Project. 2022 http://qgis.osgeo.org

32. Rousset F, genepop: Population genetic analysis using genepop. R package version 1.1.7. 2020.

33. R Core Team R: A language and environment for statistical computing. R Foundation of Statistical Computing, Vienna, Austria. 2022.

34. Waples RS. Testing for Hardy-Weinberg Proportions: Have we lost the plot?. Journal of Heredity. 2015;106: 1-19.

35. Goudet J, Jombart T, Kamvar ZN, Archer E, Hardy O. hierfstat: Estimation and tests of hierarchical F-statistics. R package version 0.5-10. 2021.

36. Foll M, Gaggiotti O. Identifying the environmental factors that determine the genetic structure of populations. Genetics. 2006;174: 875-891.

37. Nature Scot Data Analysis. [Accessed: 10.09.2021]. Available at: https://www.environment.gov.scot/data/data- analysis/

38. Marine Scotland Scotland River Temperature Monitoring Network (SRTMN) – Predictions of river temperature and sensitivity to climate change. [Accessed: 09.09.2021] Available at: https://marine.gov.scot/information/scotland-river-temperature-monitoring-network-srtmn-predictions-river-temperature-and

39. Jackson FL, Fryer RJ, Hannah DM, Millar CP, Malcolm IA. A spatio- temporal statistical model of maximum daily river temperatures to inform the management of Scotland's Atlantic salmon rivers under climate change. Science of the Total Environment. 2018;612: 1543-1558.

40. ESRI ArcGIS Desktop: Release 10. Redlands, CA: Environmental Systems Research Institute. 2011.

41. Free Map Tools, Measure a Distance. [Accessed: 12. 01. 2022] Available at: https://www.freemaptools.com/measure-distance.htm

42. Gaggiotti OE, Bekkevold D, Jorgensen HBH, Foll M, Carvalho GR, Andre C, et al. Disentangling the effects of evolutionary, demographic and environmental factors influencing genetic structure of natural populations: Atlantic herring as a case study. Evolution. 2009;63: 2939-2951.

43. Ballantyne CK, The scenery of Scotland revisited: retrospective assessment of a classic geomorphological text. Scottish Geographical Journal. 2021;137: 228-250.
44. Le Corre V, Kremer A. Cumulative effects of founding events during colonisation on genetic diversity and differentiation in an island and stepping-stone model. Journal of Evolutionary Biology. 1998;11: 495-512.
45. Eckert CG, Samis KE, Lougheed SC, Genetic variation across species' geographical ranges: the central-marginal hypothesis and beyond. Molecular Ecology. 2008;17: 1170-1180.
46. Gilbey J, Cauwelier E, Coulson MW, Stradmeyer L, Sampayo JN, Armstrong A, et al. Accuracy of assignment of Atlantic salmon (*Salmo salar* L.) to rivers and regions in Scotland and northeast England based on Single Nucleotide Polymorphism (SNP) markers. PLOS ONE. 2016;11: e0164327.
47. Harris LN, Moore JS, Galpern P, Tallman RF, Taylor EB. Geographic influences on fine-scale, hierarchical population structure in northern Canadian populations of anadromous Arctic char (*Salvelinus alpinus*). Environmental Biology of Fishes. 2014;97: 1233-1252.
48. Dillane E, McGinnity P, Coughlan JP, Cross MC, Eyto ED, Kenchington E, et al. Demographics and landscape features determine intrariver population structure in Atlantic salmon (*Salmo salar L.*): the case of the River Moy in Ireland. Molecular Ecology. 2008;17: 4786-4800.
49. Ozerov MY, Veselov AE, Lumme J, Primmer CR, "Riverscape" genetics; river characteristics influence the genetic structure and diversity of anadromous and freshwater Atlantic salmon (*Salmo salar*) populations in northwest Russia. Canadian Journal of Fisheries and Aquatic Sciences. 2012;69:1947- 1958.
50. Cauwelier E, Stewart DC. Millar CP, Gilbey J, Middlemas SJ. Across rather than between river genetic structuring in Atlantic salmon *Salmo salar* in north-east Scotland,

UK: potential causes and management implications. Journal of Fish Biology. 2018;92: 607-620.

51. Hand BK. Muhlfeld CC, Wade AA, Kovach RP, Whited DC, Narum SR, et al. Climate variables explain neutral and adaptive variation within salmonid metapopulations: the importance of replication in landscape genetics. Molecular Ecology. 2016;25: 689-705.
52. Dallaire X, Normandeau E, Mainguy J, Trembley JE, Bernatchez L, Moore JS, Genomic data support management of anadromous char fisheries in Nunavik by highlighting neutral and putatively adaptive genetic variation. Evolutionary Applications. 2021;14: 1880-1897.
53. Gilbey J, Sampayo J, Cauwelier E, Malcolm I, Millidine K, Jackson F, et al. A national assessment of the influence of farmed salmon escapes on the genetic integrity of wild Scottish Atlantic salmon populations. Scottish Marine and Freshwater Science. 2021;12: 1-70.
54. Mahlum S, Vollset KW, Barlaup BT, Skoglund H, Velle G. Salmon on the lam: drivers of escaped farmed fish abundance in rivers. Journal of Applied Ecology. 2020;58: 550-561.
55. Sylvester EVA, Wringe BF, Duffy SJ, Hamilton LC, Fleming IA, Castellani M, et al. Estimating the relative fitness of escaped farmed salmon offspring in the wild and modelling the consequences of invasion for wild populations. Evolutionary Applications. 2019;12: 705-717.

Figure.1 Click here to access/download;Figure;Figure.1.docx

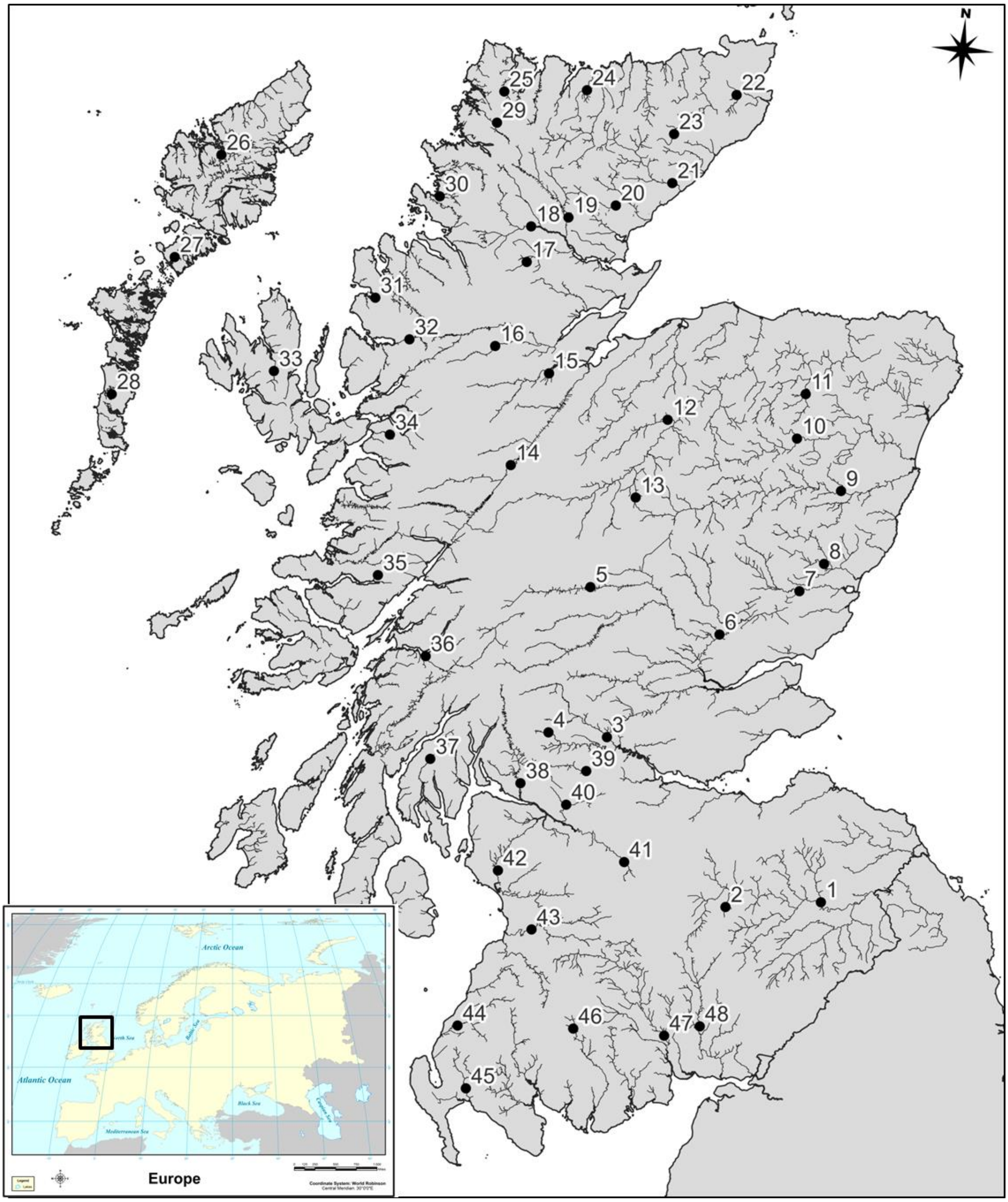

Click here to access/download;Figure;Figure.2.docx

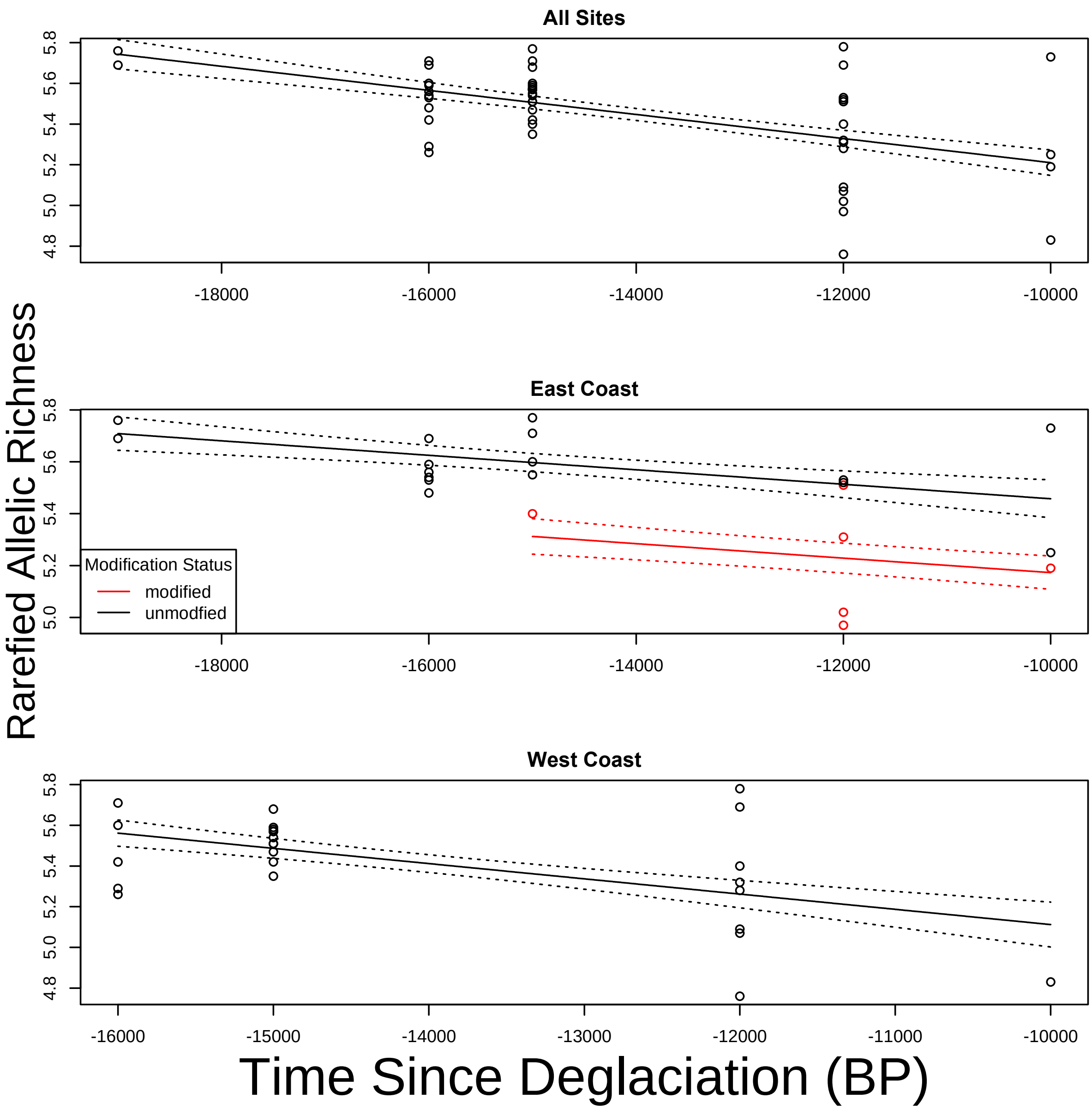

Figure.3

Click here to access/download;Figure;Figure.3.docx

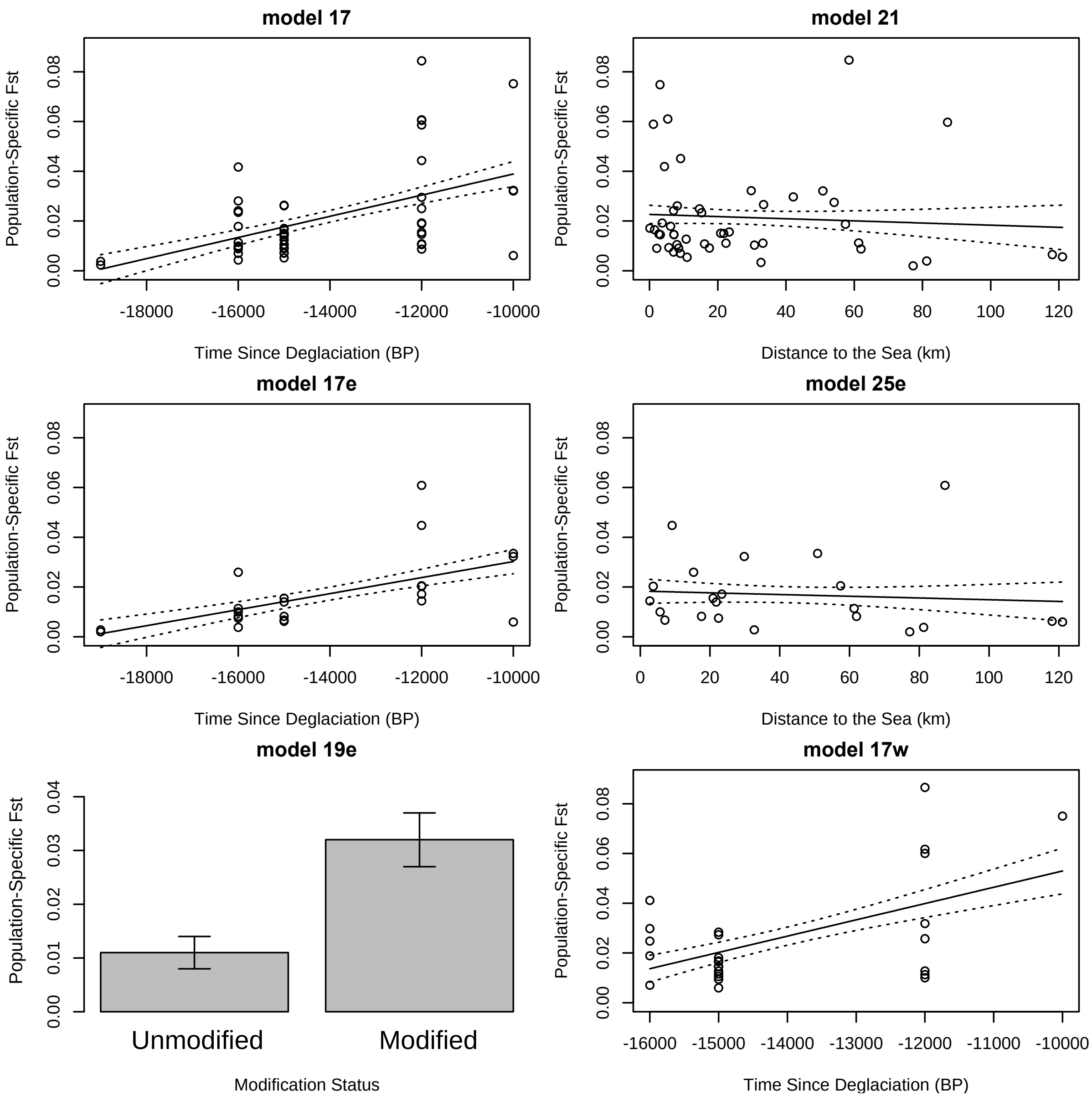

Genetic Data

Click here to access/download
Supporting Information
SeaSalar data.xlsx

Click here to access/download
Supporting Information
final supplementary material.docx
