# Supplementary material for: Post-pleistocene colonisation rather than the contemporary environment has most influenced the current population structure of Scottish Atlantic salmon (Salmo salar)
Source: PLoS One. 2025 Oct 1;20(10):e0333164. doi: 10.1371/journal.pone.0333164 (PMC12488018; doi:10.1371/journal.pone.0333164)
Supplement: Attachment — Submitted filename: PLOSONE November 2024.pdf [file pone.0333164.s004.pdf]

PONE-D-24-37086

“Post-Pleistocene Colonisation Rather than the Contemporary Environment has Most Influenced the Current Population Structure of Scottish Atlantic Salmon”

The manuscript is an interesting follow-up to Cauwelier et al. (2018). The submitted manuscript attempts to expand on the previous report on Atlantic salmon population structure in Scotland. I can understand why the authors invested time and resources into the new study.

1) Unfortunately, the metrics selected to study simply don’t have enough variability to build meaningful models. Two metrics are class/categorical variables. The temperature data varies by only a few degrees. The river length were short in terms of distance salmon travel to reach spawning sites; most of these distances could be travelled in a day or two and therefore it is not a real measure of a distance challenge that might be a predictor of population structure. As an aside, the authors don’t show what appears to be nine (9) metrics in a list or summary table. Related to this is the metric “elevation” which is mentioned but does appear again; river slope might be useful metric to add.
2) The distance/connectivity is problematic, “population-specific measure of connectivity”, because distance is distance. By combining these two measures, the larger number (between rivers) most probably overrides the biological relevance of the in-river distance.
3) Selecting 48 of the original salmon rivers was described as a random process with no further clarification. The original study demonstrated that the overall group of Scottish salmon is partitioned into several definable units. The authors don’t mention how you addressed the potential impacts of a simple random selection from classes of data.
4) There are couple of editorial comments in the manuscript you may find useful for future writing. For example, melting ice sheets (glaciers) aren’t actually moving, just melting. If you were invoking the movement effect as a metric of interest, then you would need to demonstrate that glaciers were surging and concurrently varying accessibility by salmon to the rivers.

Overall, the problem with the lack of variability within metrics is demonstrated in the statistically, non-significant models. A different set of metrics, each with reasonable variability among sites might help to tease out the role of the contemporary environment.
