# Supplementary material for: Post-pleistocene colonisation rather than the contemporary environment has most influenced the current population structure of Scottish Atlantic salmon (Salmo salar)
Source: PLoS One. 2025 Oct 1;20(10):e0333164. doi: 10.1371/journal.pone.0333164 (PMC12488018; doi:10.1371/journal.pone.0333164)
Supplement: Attachment — Submitted filename: Reviewer 2 - response to Authors Jan 2025.pdf [file pone.0333164.s006.pdf]

Second Review of "Post-Pleistocene Colonisation Rather than the Contemporary Environment has Most Influenced the Current Population Structure of Scottish Atlantic Salmon (*Salmo salar*)" by Cowell *et al*.

ORIGINAL REVIEW - *Unfortunately, the metrics selected to study simply don't have enough variability to build meaningful models. Two metrics are class/categorical variables. The temperature data varies by only a few degrees. The river length were short in terms of distance salmon travel to reach spawning sites; most of these distances could be travelled in a day or two and therefore it is not a real measure of a distance challenge that might be a predictor of population structure. As an aside, the authors don't show what appears to be nine (9) metrics in a list or summary table. Related to this is the metric "elevation" which is mentioned but does appear again; river slope might be useful metric to add.*

Response:

This reviewer takes issue with an apparent lack of variability in some of the metrics we used. They note our use of categorical variables but do not explain why this results in a lack of variability. We acknowledge in our manuscript that the use of these variables has limitations (as do all data) but we feel the use of standardised metrics of water quality and waterbody modification are justified in their use in this study. The reviewer takes further issue with our use of temperature data that they have decided does not vary enough. While we can certainly imagine circumstances under which higher temperatures could have a greater impact than the temperatures in the rivers we are studying, we are using actually existing data so our conclusions are that the temperatures that our rivers have experienced are not having a major impact. Given the data, this is a valid conclusion and does not say anything about, for example, if temperatures were to rise further due to our climate change.

A major misunderstanding seems to have taken place regarding the distance to the sea measures we used. Firstly, Scottish rivers are only so long, meaning that, when investigating distance to the sea effects in Scotland, it is a simple reality that distances will be shorter compared to other parts of the world. Within Scotland, our sites certainly include the upper reaches of the major drainage systems. Secondly, distance to the sea (as explained in the introduction) is of interest to us due to the effect this may have had on the colonisation process, not because the distances considered are in some way too far for a salmon to travel. Thirdly, altitude was not included in the final analysis due to it being closely correlated with distance to the sea and therefore rising above our correlation coefficient and VIF thresholds. We have now clarified this on lines 190-191 of our manuscript.

To summarise our thoughts, we believe that this reviewer is asking us to find levels of variation that do not exist between Scottish salmon populations.

Reviewer #2

2) The distance/connectivity is problematic, "population-specific measure of connectivity", because distance is distance. By combining these two measures, the larger number (between rivers) most probably overrides the biological relevance of the in-river distance.

Response:

We refer to our response to reviewer 1 when it comes to explaining our use of population-specific measures. Although "distance is distance", the effect of genetic differentiation between pairs of populations is mediated not only by distance between focal populations but also by the exchange of migrants with other local populations and by differences in local population size; as we explain above.

We refer to our earlier response to reviewer 2 which clarifies that distance to the sea is not being used as a measure of contemporary connectivity. Although we appreciate that other authors have used this measure as a proxy for connectivity, these tend to be employed for populations with rivers draining to a single coastline, or with populations whose rivers flow into the same sound, for example. In our case we consider rivers which drain to different coasts of the British Isles so distance to the sea would be a very poor measure of connectivity. Our results illustrate the difference between distance to the sea and connectivity very well, with connectivity being shown not to be of great importance, while distance to the sea is present in many of the diversity and differentiation models. This is in fact the opposite of what this reviewer seems to think would happen. Across all sites and on the east coast, distance to the sea accompanies time since deglaciation, highlighting that distance to the sea is of relevance to the colonisation process.

Reviewer #2

Overall, the problem with the lack of variability within metrics is demonstrated in the statistically, non-significant models. A different set of metrics, each with reasonable variability among sites might help to tease out the role of the contemporary environment.

Response:

We strongly disagree with this interpretation of our results. Statistically significant variables were identified as having an influence on genetic diversity. This was not only the case for time since deglaciation, but also waterbody modification on the east coast (a categorical variable). Our GESTE results allude to distance to the sea having some importance as well, despite this reviewer's claim that there is insufficient variability for this metric to be useful. The variables found to not have a statistically significant effect are a reflection of the reality of the sites we included in this study, which, as discussed, give good geographical and genetic coverage of Scotland's salmon rivers, as identified by Cauwelier et al. (2018).
