# Supplementary material for: Post-pleistocene colonisation rather than the contemporary environment has most influenced the current population structure of Scottish Atlantic salmon (Salmo salar)
Source: PLoS One. 2025 Oct 1;20(10):e0333164. doi: 10.1371/journal.pone.0333164 (PMC12488018; doi:10.1371/journal.pone.0333164)
Supplement: Attachment — Submitted filename: Response to Reviewers.docx [file pone.0333164.s008.docx]

Reply to editor

It is my pleasure to inform you that the review process of your MS has completed. I was glad that two expert in the field of fish population genetics accepted our invitation to review your submission. They found your research very interesting, however had some concerns regarding the methods (particularly genomic analysis and and variables) to investigate population differentiation and diversity of Atlantic salmon in Scotland. Here, I would like to ask you revising your MS with careful consideration to the comments provided by reviewers, and provide them with point-by-point responses to the comments. Considering the comments, your revision will be subjected to another peer-review

We are grateful for the effort on the part of our reviewers to provide us with comments, some of which are extremely helpful. We have accepted many of their recommendations, such as making changes to figures and making stylistic changes to the writing, improving the clarity of the manuscript. There are however some major issues that both reviewers took with regard to our methodology. We disagree with the premise of these criticisms and address them fully.

5. Review Comments to the Author

Please use the space provided to explain your answers to the questions above. You may also include additional comments for the author, including concerns about dual publication, research ethics, or publication ethics. (Please upload your review as an attachment if it exceeds 20,000 characters)

Reviewer #1: I have now read the manuscript “Post-Pleistocene Colonisation Rather than the Contemporary Environment has Most Influenced the Current Population Structure of Scottish Atlantic Salmon (Salmo salar)” In this study, the authors expand on a microsatellite panel for a subset of Atlantic Salmon populations previously studied in Scotland. They conclude that the Time since deglaciation is the major determinant for diversity and differentiation in those populations.
I want to start my review by stating that I have never directly worked with microsatellite data, as my expertise is leaning toward genomic analyses (both GBS and low coverage WGS). That being said, I was excited to see this enormous dataset revisited with such interesting questions as the determinants (historical vs contemporary) of genetic diversity. However, I wonder if this was the best choice to reduce the number of populations to increase the number of markers, as the near-exhaustive sampling of Scottish salmon populations in Cauwelier et al. 2018 was very impressive and could have yielded interesting insight into those questions by controlling for within and between watershed effects. However, I understand that it is unreasonable to ask the authors to revisit this design choice.
Rather, my main concern about this manuscript is the treatment of population differentiation, which is central to the study. In the current version, differentiation is treated as an average of the pairwise Fst of a focal population with all others present in the dataset. To me, this measure has little biological meaning and is very dependent on the populations included in the dataset.
As such, I believe the authors should consider reworking considerably the portions of their study related to differentiation. There is abundant literature on isolation-by-distance driving patterns of differentiation in salmonids, and right now I feel neither connectivity nor distance by sea is properly accounting for this. I suggest that the differentiation should be analyzed using pairwise Fst (by pair of populations), rather than ‘population-specific Fst’. This way, the author could introduce either the marine distance between river mouths or the total distance between sampling sites to first control for isolation-by-distance. Then, additional variables, such as the difference in timing of deglaciation between the 2 focal populations could be introduced as covariable, i.e. to test if two populations are more similar than expected given the geographical distance separating them if they occupy habitats that became free of ice at the same time. Other covariables could be used in a similar fashion.
In light of this, my assessment of the present manuscript is that it needs a major revision of the central analyses in order to yield insight in the questions proposed by the authors. I have attached below some more specific comments.

We are grateful for many of this reviewer’s comments and the time they clearly put into reading our manuscript. While we may not be able to allay all of their misgivings, we believe that changes to our manuscript will be helpful to future readers.

This reviewer believes that the population-specific measures of Fst used in this study have little biological meaning. We strongly disagree with this idea as population-specific Fst is strongly grounded in fundamental concepts in metapopulation biology and population genetics. Moreover, it can be argued that, despite being widely used, pairwise Fst measures are ad-hoc and lack theoretical grounding. The study of isolation by distance using pairwise Fst relies on two completely unrealistic assumptions:

1) Differentiation between a pair of populations is not influenced by the exchange of migrants that the two focal populations may have with other local populations. In other words, the fact that differentiation between a pair of populations can be influenced by exchange of genetic material those two populations have with other local populations can be completely ignored.
2) Differences in local population size has no effect of genetic differentiation between the pair of populations. This assumption ignores the fact that differences in population size also determine differences on the effect of genetic drift on genetic differentiation.

Clearly these two assumptions contradict standard population genetic principles stating that both genetic drift and migration influence genetic differentiation in a spatially-structured population.

On the other hand, the population-specific Fst concept explicitly acknowledges the fact that both global patterns of migration as well as local population size influence genetic differentiation between populations, as explicitly stated by population genetics theoretical principles that can be found in any introductory population genetics textbook. Moreover, it is strongly grounded on the concept of population connectivity, as developed in the domain of metapopulation biology. Indeed, any meaningful measure of isolation needs to take into account both distance and size of the local populations (see Box 11.1 in Hanski 1999; for a more advanced treatment of this issue see Hanski & Gaggiotti 2004).

All the above theoretically- and conceptually-grounded arguments justify the use of local population Fst while disqualifying the use of pairwise Fst. We need to point out that the fact that a measure or statistic is widely used does not imply that it is appropriate or biologically meaningful.

In addition to the theoretical support for population-specific measures of Fst, and its ability to detect any existing IBD, while considering global migration patterns and differences in local population size, we are also sure that an IBD signal would not in fact be detected using pairwise measures of Fst and distance in this instance. This is because we had previously conducted a classic mantel test using these metrics. The result of this test corroborated our result using population-specific measures. We have now included a description of the mantel test methodology (lines 211-215) and the associated result (lines 263-264). It is however worth pointing out that we prefer the use of population-specific variables as supported by the theoretically-grounded arguments we outline above.

METHODS
Ln 175: It is rather unclear what Connectivity represents in this context, especially since your dataset does not pretend to encompass every salmon population in the region, as you specifically used a portion of those studied in Cauwelier et al. 2018. As used presently, the measure would be more meaningful if estimated using every known Scottish population, but even then, I feel like the pairwise river+marine distances would be better used in an isolation-by-distance analysis, as I discussed above.

Our study uses connectivity between sites to investigate if such a pattern is present between our sampling populations. The sites used in this study are a subset of those used in Cauwelier *et al.* (2018) and these encompass the major clustering groups identified in 2018, giving us good geographical coverage as a result. We therefore feel confident in using connectivity (which we clearly describe in lines 183-185 as the mean of all pairwise distances between a population and all other populations) as a measure. This is a method that has been applied before in highly cited publications (e.g., Foll & Gaggiotti, 2006; Gaggiotti *et al.,* 2009; Hand *et al.,* 2016; Kittlein & Gaggiotti, 2008). In addition, we have now included the mantel test results, corroborating our population-specific results (lines 263-264).

RESULTS
Ln 215-216: Is this positive or negative? What does this mean concerning the filtering? That no markers were removed?
Suggestion: As Hardy-Weinberg equilibrium deviation and positive tests for LD were not significantly greater than the number expected due to effects of chance alone, all 33 microsatellite loci were analyzed.

Done.

Ln 216: Suggestion for clarity: Average rarefied allelic richness across the 48 Scottish Atlantic Salmon populations equaled 5.47 (SD = 0.25).

Done, except that we retain the word mean, rather than use the word average.

Ln 218: Add the population number (in parentheses) as in Fig. 1 to the river name, as this is much easier to follow.

Done.

Ln 239: This whole sentence lacks in readability. It should also be clear that we moved on from diversity to differentiation.

We have changed this sentence so that it is now clear that we are talking about genetic differentiation (lines 258-260).

Ln 242: ‘Non-negligeable’ : Did you use a specific threshold?

No, this was very much an interpretation based on the fact that the marginal posterior probabilities were 0.524, 0.162 and 0.144 for time since deglaciation, waterbody modification and distance to the sea, respectively. The next highest was 0.044 and we considered this to be low enough not to discuss in detail, but we do not of course consider these variables to have no biological meaning, only that their effect on genetic differentiation is quite small. One of the advantages of using a Bayesian analysis is that it allows us to consider more nuance in the results by not using fixed thresholds. In order to clarify this, we have changed the wording from “non-negligible” to “relatively high” (line 262).

DISCUSSION
Ln 300-301: Could you please provide reference for this claim? It seems to be very specific to anadromous fish in this specific landscape.

This result has been found in two separate studies on different parts of the east coast of Canada (Dionne *et al.,* 2008; Harris *et al.,* 2014). These references are cited in the previous sentence, in the previous paragraph. We take this opportunity to rewrite this sentence explaining why one might expect a positive relationship between genetic differentiation and distance to the sea (lines 322-324).

Ln 304-306: Assuming we are talking about anadromous populations of salmons, I’m not sure I would refer to dispersal inside a watershed as colonization. Can we really expect a strong founder effect as fish that migrate along a river start spawning in newly available habitats inside their natal watershed? I wonder, but most generally I feel that the implications of the anadromy inside the studied populations are not sufficiently discussed in this paper, both in the introduction and discussion.

The time between the lower section of the watershed and the upper sections of the watershed becoming ice free numbers in thousands of years. If colonisation occurred downstream, this would also be expected to be a lengthy process owing to the slow nature of isostatic rebound. We therefore feel that the use of the term colonisation is justified.

FIGURES
Fig. 1: Could you add the genetic clustering information referred to in the text (Cauwelier et al. 2018), or at the very least the membership to either the West or East group relevant to the hierarchical analysis?

Done.

Fig. 2: This is rather stylistic note, but I feel the trends would be more apparent and comparable if the plots were closer to a square in shape, as there is not a lot of density of points to show on the x-axis. That would also allow the three panels to by side-by-side, so we can better appreciate the differences in allelic richness in the west and east. For the same reason, I also suggest that you use the same extent (min and max) on both the x- and y-axis for all three plots.

Done.

This would also allow you to add a second line of plots to show the effect of distance to sea (all sites + east), which I don’t understand the absence in Figures. This would help to support the claims made at Ln 278-279.

For our genetic diversity analysis, using rarefied allelic richness, we used a frequentist approach. The effect of distance to the sea was not statistically significant (see table 1) and therefore we did not include it in the plots. The model does however contain distance to the sea as a term in the model (as chosen by AIC) and therefore the plots in figure 2 show the results of the significant variables, accounting for the effect of distance to the sea. We have added an extra sentence into the figure legend in order to clarify this.

Fig. 3: Similar to my comment on Fig. 2, I suggest that the panels be reorganised so the reader can more visually compare the results of different model and region.

Done

Reviewer #2: See attachment.

PONE-D-24-37086
“Post-Pleistocene Colonisation Rather than the Contemporary Environment has Most Influenced the Current Population Structure of Scottish Atlantic Salmon”

The manuscript is an interesting follow-up to Cauwelier et al. (2018). The submitted manuscript attempts to expand on the previous report on Atlantic salmon population structure in Scotland. I can understand why the authors invested time and resources into the new study.

We are grateful for this reviewer for their helpful comments. I believe our manuscript now reads more clearly as a result. The reviewer clearly has several misgivings regarding the data that we used and we address this issue in our response.

1) Unfortunately, the metrics selected to study simply don’t have enough variability to build meaningful models. Two metrics are class/categorical variables. The temperature data varies by only a few degrees. The river length were short in terms of distance salmon travel to reach spawning sites; most of these distances could be travelled in a day or two and therefore it is not a real measure of a distance challenge that might be a predictor of population structure. As an aside, the authors don’t show what appears to be nine (9) metrics in a list or summary table. Related to this is the metric “elevation” which is mentioned but does appear again; river slope might be useful metric to add.

This reviewer takes issue with an apparent lack of variability in some of the metrics we used. They note our use of categorical variables but do not explain why this results in a lack of variability. We acknowledge in our manuscript that the use of these variables has limitations (as do all data) but we feel the use of standardised metrics of water quality and waterbody modification are justified in their use in this study. The reviewer takes further issue with our use of temperature data that they have decided does not vary enough. While we can certainly imagine circumstances under which higher temperatures could have a greater impact than the temperatures in the rivers we are studying, we are using actually existing data so our conclusions are that the temperatures that our rivers have experienced are not having a major impact. Given the data, this is a valid conclusion and does not say anything about, for example, if temperatures were to rise further due to our climate change.

A major misunderstanding seems to have taken place regarding the distance to the sea measures we used. Firstly, Scottish rivers are only so long, meaning that, when investigating distance to the sea effects in Scotland, it is a simple reality that distances will be shorter compared to other parts of the world. Within Scotland, our sites certainly include the upper reaches of the major drainage systems. Secondly, distance to the sea (as explained in the introduction) is of interest to us due to the effect this may have had on the colonisation process, not because the distances considered are in some way too far for a salmon to travel. Thirdly, altitude was not included in the final analysis due to it being closely correlated with distance to the sea and therefore rising above our correlation coefficient and VIF thresholds. We have now clarified this on lines 190-191 of our manuscript.

To summarise our thoughts, we believe that this reviewer is asking us to find levels of variation that do not exist between Scottish salmon populations.

2) The distance/connectivity is problematic, “population-specific measure of connectivity”, because distance is distance. By combining these two measures, the larger number (between rivers) most probably overrides the biological relevance of the in-river distance.

We refer to our response to reviewer 1 when it comes to explaining our use of population-specific measures. Although “distance is distance”, the effect of genetic differentiation between pairs of populations is mediated not only by distance between focal populations but also by the exchange of migrants with other local populations and by differences in local population size; as we explain above.

We refer to our earlier response to reviewer 2 which clarifies that distance to the sea is not being used as a measure of contemporary connectivity. Although we appreciate that other authors have used this measure as a proxy for connectivity, these tend to be employed for populations with rivers draining to a single coastline, or with populations whose rivers flow into the same sound, for example. In our case we consider rivers which drain to different coasts of the British Isles so distance to the sea would be a very poor measure of connectivity. Our results illustrate the difference between distance to the sea and connectivity very well, with connectivity being shown not to be of great importance, while distance to the sea is present in many of the diversity and differentiation models. This is in fact the opposite of what this reviewer seems to think would happen. Across all sites and on the east coast, distance to the sea accompanies time since deglaciation, highlighting that distance to the sea is of relevance to the colonisation process.

3) Selecting 48 of the original salmon rivers was described as a random process with no further clarification. The original study demonstrated that the overall group of Scottish salmon is partitioned into several definable units. The authors don’t mention how you addressed the potential impacts of a simple random selection from classes of data.

We have now clarified that numbers of sites selected were proportional to each clustering group (lines 117-121).

4) There are couple of editorial comments in the manuscript you may find useful for future writing. For example, melting ice sheets (glaciers) aren’t actually moving, just melting. If you were invoking the movement effect as a metric of interest, then you would need to demonstrate that glaciers were surging and concurrently varying accessibility by salmon to the rivers.

All references to moving ice sheets have been changed to melting.

Overall, the problem with the lack of variability within metrics is demonstrated in the statistically, non-significant models. A different set of metrics, each with reasonable variability among sites might help to tease out the role of the contemporary environment.

We strongly disagree with this interpretation of our results. Statistically significant variables were identified as having an influence on genetic diversity. This was not only the case for time since deglaciation, but also waterbody modification on the east coast (a categorical variable). Our GESTE results allude to distance to the sea having some importance as well, despite this reviewer’s claim that there is insufficient variability for this metric to be useful. The variables found to not have a statistically significant effect are a reflection of the reality of the sites we included in this study, which, as discussed, give good geographical and genetic coverage of Scotland’s salmon rivers, as identified by Cauwelier *et al.* (2018).

References

Cauwelier, E. Verspoor, E. Coulson, M. W. Armstrong, A. Knox, D. Stradmeyer, L. Webster, L. M. I. and Gilbey, J. (2018) Ice sheets and genetics: insights into the phylogeography of Scottish Atlantic salmon, *Salmo salar* L.. *Journal of Biogeography,* 45: 1-13.

Dionne, M. Caron, F. Dodson, J. J. and Bernatchez, L. (2008) Landscape genetics and hierarchical genetic structure in Atlantic Salmon: the interaction of gene flow and local adaptation. *Molecular Ecology,* 17: 2382-2396.

Foll, M. and Gaggiotti, O. (2006) Identifying the environmental factors that determine the genetic structure of populations. *Genetics,* 174: 875-891.

Gaggiotti, O. E. Bekkevold, D. Jorgensen, H. B. H. Foll, M. Carvalho, G. R. Andre, C. and Ruzzante, D. E. (2009) Disentangling the effects of evolutionary, demographic and environmental factors influencing genetic structure of natural populations: Atlantic herring as a case study. *Evolution,* 63: 2939-2951.

Hand, B. K. Muhlfeld, C. C. Wade, A. A. Kovach, R. P. Whited, D. C. Narum, S. R. Matala, A. P. Ackerman, M. W. Garner, B. A. Kimball, J. S. Stanford, J. A. and Luikart, G. (2016) Climate variables explain neutral and adaptive variation within salmonid metapopulations: the importance of replication in landscape genetics. *Molecular Ecology,* 25: 689-705.

Hanski, I. (1999) Metapopulation Ecology. Oxford University Press, 313pp.

Hanski, I. and Gaggiotti O.E. (2004) , *Ecology, Genetics, and Evolution of Metapopulations*. Elsevier-Academic Press, 696pp.

Harris, L. N. Moore, J. S. Galpern, P. Tallman, R. F. and Taylor, E. B. (2014) Geographic influences on fine-scale, hierarchical population structure in northern Canadian populations of anadromous Arctic char (*Salvelinus alpinus*). *Environmental Biology of Fishes,* 97: 1233-1252.

Kittlein, M. J. and Gaggiotti, O. E. (2008) Interactions between environmental factors can hide isolation by distance patterns: a case study of *Ctenomys rionegrensis* in Uruguay. *Proceedings of the Royal Society B,* 275: 2633-2638.
