# Supplementary material for: Post-pleistocene colonisation rather than the contemporary environment has most influenced the current population structure of Scottish Atlantic salmon (Salmo salar)
Source: PLoS One. 2025 Oct 1;20(10):e0333164. doi: 10.1371/journal.pone.0333164 (PMC12488018; doi:10.1371/journal.pone.0333164)
Supplement: Attachment — Submitted filename: Response_to_reviewers_auresp_2.docx [file pone.0333164.s009.docx]

Reply to editor

Dear Finn Cowell; The review of revised MS you submitted to PLOS ONE has completed. To reach the current decision, we invited additional reviewers to review the revised MS. Referees provided us with very valuable comments, I warmly appreciate for their agreement to review this submission and for very valuable comments. I would like to ask you revising your MS for another time according to the comments appended below. In addition to the referees’ comments, please consider those of mine in your revision:

1- I agree with R#4 to re-locate Table S1 in the main body of the paper.
2- Describe the methods in detail.
3- Follow the guide from PLOS ONE for the use of map: it needs to indicate copyright.
4- For figures 2 and 3: Increase the font size for numbers shown in X-axis and Y-axis as well as title for each panel.

We are grateful for the feedback received on this submission and we have altered our manuscript to comply with the above comments. We thank both yourself and the reviewers for their time and effort and have made a number of changes to the manuscript, as well as responding to all the reviewers’ comments.

Reviewer #1: I enjoyed reading the manuscript for a second time, with a bit more perspective. As stated in my original review, I was less familiar with some of the methods and approaches used here, so I apologize to the authors if my original review lacked nuance at times. I want to thank them for their enlightening, if not humbling, response.

I consider this study to offer a valuable contribution to the field, and now that my concerns have been addressed, I recommend its publication in its current form.

We take this opportunity to once again thank this reviewer for their initial comments, many of which helped us to greatly improve the manuscript. We are glad to hear that our responses were interesting.

Reviewer #2: I have enclosed more comments in two files. One is a response to your response to my review and the other provides some additional commentary on your manuscript (I used the section with your original manuscript and your responses to the reviews). My fundamental criticism was that your selected metrics don’t have the variability necessary to tease out the mechanisms of population structure. Your overall goal and the methods are not at issue. You have a great looking genetics data set and I am confident that environment metrics exist that can help delineate mechanisms of structuring.

We thank this reviewer for their continued engagement with our manuscript and attempt to address the issues raised by them. We respectfully disagree with this reviewer’s central claim, which is that the metrics we use lack sufficient variability. We go through the comments in response to our manuscript followed by their response to our initial response. The line numbers we have inserted as part of the comments that reviewer two made on the marked up version of the manuscript.

Lines 117-121: Clarity - It appears there are 7 groups and 48 sites. There are differences in the number of rivers/populations within each group (presumably) - tell us how you ensured that no group was over represented in the analyses, e.g., if there there had been 4 genetic groups (clusters of rivers/populations), then you sampled n=12 from each group and these 12 were randomly selected within a group (n = 48).

Some of the genetic clusters encompassed only a few sampling sites over a very limited geographic area. An equal number of sites per genetic cluster would therefore have resulted in a very small geographic area being overrepresented. We therefore had to sample from all genetic clusters, ensuring sites were not geographically concentrated, and the sites chosen are a reflection of our attempts to strike this balance.

Lines 194-195: The inclusion of elevation is important in a metric of river slope that reflects the probability of accessing the upper reaches, i.e., as important as distance, which needs to be included, assessed, and dismissed if that is the finding.

Given that the fundamental criticism from this reviewer is that our metrics are not variable enough, we now consider if the proposed alternative of river slope is in fact more variable than distance to the sea. The coefficient of variation for distance to the sea is equal to 0.9, while for river slope this only equals 0.486. The variance of the logarithm of distance to the sea is 1.91, while for river slope this same measure of variability is equal to 0.756. Lewontin (1966) notes a ratio of the latter metric can be compared to the F distribution. In this case the F value at 47 degrees of freedom, and using 0.05 as the significance threshold, is less than the ratio of 2.53 which we calculated. Therefore, not only are these measures of variability showing that distance to the sea is more variable than the river slope, our F-test suggests that it is significantly more variable. We do not therefore think it is worth rerunning a computationally taxing GESTE run to include a less variable metric, when low variability is the chief concern of this reviewer.

We will also take this opportunity to point out that we do not claim that this study has considered all possible metrics of interest. We do however consider a great many, and from our perspective, the statistical effect (small or large) that each of these have on genetic diversity and differentiation is of interest, even if other influences are yet to be understood.

Lines 306-308: Here is an example of the over-interpretation of results. There is no evidence in this study that waterbody modification had an "effect". You did see a correlation between these observations that suggest a relationship, but without a controlled experiment you can't know its an effect, and additionally, "non-negligible" is not the correct term, "apparent" is appropriate in all of our field studies.

We respectfully disagree that we are overinterpreting our results because we are not referring to a causal effect. It is common practice to use the word effect to describe a statistical effect. Michael Morrissey’s book *Elementary Statistical Modelling* has the following words to say on the subject:

*‘ In every day usage, the wording “A has an effect on B” may well be taken to mean A has a causal effect on B. This very strong meaning is not typically intended when people use the word “effect”. This is just typical terminology typically used by biologists to describe different kinds of associations between variables (e.g., as can be expressed by the slope of a regression line). ‘*

Line 323: The alternative interpretation as I first suggested is that your metrics don't have the variability necessary to expose mechanisms; the class variables are too coarse (as you recognize) and your numerical metrics vary too little. In both cases this impacts the mathematics (the strength of your predictability which depends on variation) and the biological relevance of the selected metrics, i.e., the animals' range of tolerances (their behaviour over time) likely exceed the environmental variation you are using in the analyses (the temperature comment in my first review).

This comment is made in response to our distance to the sea section of the discussion and we believe we have addressed the fact that the river slope is less variable than distance to the sea. We are sure that some of the class variables we have used might have information underlying them that would be of interest. The class variables were however obtained from databases created by researchers with expertise in environmental monitoring. As such they encapsulate all the variables that were considered as relevant for describing the extent to which the river has been affected by human activities. We therefore expect a waterbody with good water quality to be different from one categorised as bad. We also expect heavily modified water bodies to differ from those that are not, although we admit that the way in which water bodies are modified will vary.

Finally, this reviewer takes issue with our temperature metric as the temperatures do not exceed the animals’ range of tolerances. We used the maximum daily temperature on the hottest year for the last 20 years. This is a metric that the Scotland River Temperature Monitoring Network (SRTMN) made available and we do not know of any other source of nation-wide river temperature estimates for specific stretches of river that are reliable. The maximum temperature over a shorter time period than a day is not something made available by the SRTMN so we believe this is the best metric that can be used. Finally, we need to stress that the rationale for the choice of maximum temperature is that extreme events can cause events like major fish mortality or inaccessibility to sections of river, as hypothesised in Hand *et al.* (2016).

We mention in our discussion that previous studies have shown increased genetic differentiation in areas of high temperature, while others show reduced differentiation. A lack of variability is therefore not the only explanation for temperature not being found to greatly influence genetic differentiation. The causal mechanism might not be consistent. Consider the hypothesising done by Hand *et al.* (2016) in their population genetic study of anadromous *Oncorhynchus mykiss.* They considered the possibility that ‘ *Higher stream temperatures lead directly to increased mortality or indirectly through increased physiological stress during return migration for adults. Thus, increased mortality leads to fewer returning breeders and lower local Ne* ‘ while also acknowledging the alternative possibility that ‘ *Higher stream temperatures lead to blocked routes of migration to natal streams further increasing straying rates leading to lower FST* ‘. That is to say, the first biological mechanism of Hand *et al.* (2016) might be at work in some areas, while the other mechanism might be counterpoising this in other areas. Incidentally, Hand *et al.* (2016) used maximum daily temperatures as well*.*

Lines 324- 340: This is quite a bit of logic gymnastics to explain your results which as I have tried to point out, remain inconclusive with respect to your recolonization progression question.

This section of the discussion may well be too long and, since two reviewers drew our attention to this section, with reviewer 4 suggesting that we cut it down, we do so. We appreciate that there remain some unexplained results, but we do not believe this to be a major issue as this study is not all encompassing.

Line 363: correlation?

This section has now been removed from this discussion after considering feedback from both reviewers 2 and 4.

Line 379-383: Here is an example of the coarseness of scale affecting your results and interpretation. It is the efficiency of fish passage that will impact genetic structure as many studies demonstrate. That is the metric that would have given you the power of resolution you are seeking.

We consider this to be a surprisingly difficult variable to measure in a quantitative way. Firstly, hydro schemes do not simply act as a barrier that obstruct upstream passage. For example, they also abstract water which reduces the carrying capacity of the river below a dam and this is likely to have an impact on genetic diversity and differentiation. This is relevant for several of the sites we consider. Some dams are also more easily passed than others but quantifying this may be difficult.

Another consideration to factor in is historical stocking that occurred above dams in Scotland and continues to occur in the form of mitigation stocking. Poor records make it almost impossible to consider the impact of the former, while the latter is also difficult as brood stock are often caught afresh each year and this likely involves capturing individuals which originated from elsewhere in the catchment, increasing gene flow. This could counterpose the reduced gene flow from the barriers. We therefore think that, given current knowledge, any measure of passage efficiency would be unreliable and likely to introduce biases in our analyses.

Line 416: This is the reason to include a metric like river slope which was available to you.

We address this above where we show that river slope is less variable than distance to the sea.

Reviewer #2 response to our initial response.

The lack of variability isn’t ‘apparent’ it is obvious, and the authors make reference to it throughout.

We suggest that these are limitations that are well within the realms of acceptability. Often the reduced variability that we make reference to is a feature of the landscape that these fish occupy, such as the reduced distance from the sea on the west coast compared to the east coast. In other words, Scottish rivers and streams do not exhibit much variation in the environmental variables to which we had access.

As the authors point out, categorical variables by there very nature mask the natural variation in the parameters they represent.

Categorical variables mask some of the variation but certainly not all of it. We do not believe that this restriction in variability results in there being no utility to using these categorical variables.

I agree that these standard metrics are used and useful, but they mask the variation in the natural environment that are the mechanism of selection and population structure the authors seek to explain.

This is again a limitation that we understand, and which biologists for hundreds of years have understood, and this has not stopped us, or the biologists down the years, from utilising categorical variables. We do not understand why, in our case, it is wrong for us to utilise categorical variables.

To the authors – this type of passive aggressiveness doesn’t help your argument. In this case, I have not decided anything. I have pointed out that the temperature data (the metric you selected) varies too little to be a useful metric – mathematically and biologically. You conclude that your temperature metric provided no support for population structuring and you correctly point out that temperature is a critical aspect of structuring. My comment is unchanged: river temperature is important, but the metric selected doesn’t have the variability necessary to help discern underlying mechanisms of population structuring.

Firstly, we would like to apologise for any offence caused to this reviewer and can say with all honesty that no passive aggressive tone was intended in what was a clumsily worded sentence on our part. We do however respectfully disagree with this reviewer that our temperature metric is inappropriate and we have hopefully addressed this above to some extent. We will however add here that, although we write about the impact of temperature in other population genetic studies, it does not logically follow that we are going to find a similar result in different populations experiencing a different temperature regime.

Another note to the authors. You have incorrectly assumed that I don’t know Scottish rivers and their landscapes.

Apologies.

I used the distance a salmon can travel comment to point out that this metric is more nuanced than distance alone, e.g., river slope, numbers and location of natural/human barriers. You haven’t captured these elements of the riverscape by distance alone.

We have addressed some of these points above, but we would like to stress here that our study is not exhaustive and we chose variables based upon what we thought might have an effect on genetic diversity and differentiation, which in turn was based on previously published papers.

I wasn’t clear the first time – altitude gives you a river slope which is an important metric you could have included.

Now understood and hopefully addressed above.

I am not asking you to find variation where it doesn’t exist; I am pointing out that there are metric with better resolution that will give you the variability necessary to tease out the mechanisms of population structuring.

We are interested in the metrics we chose for this study, as the introduction to our manuscript justifies. The variability of the other metrics proposed by this reviewer have limitations of their own which we have paid attention to in various places above.

My comments remain the same. Your models lack parameters with enough variation to meaningful discuss their potential connection to mechanisms of population structuring. For example, the time since deglaciation has 4 moments of time and in each figure the predicted relationship is the result of 2-3 of 48 points. You also point out that outliers are an issue in your analyses.

We would not feel comfortable considering more specific estimates of the deglaciation process than those created by Cauwelier *et al.* (2018). Considering a process that took place thousands of years ago is always going to be difficult to measure very precisely. Outliers are a perfectly ordinary thing to discover when analysing data collected from wild populations. Understanding these is important but an outlier is not an invalid data point simply by virtue of being an outlier.

Again, my comments reflect your choice of metrics which lack variability. There are most probably metrics that could tease out the mechanism you are seeking, but the metrics selected don’t have that power, neither mathematically or biologically.

We believe we have addressed this comment already: the metrics we considered were found to be of interest by previous studies but they have limited variability across Scottish river drainages. This is a fact that we cannot change/ignore.

Reviewer #3: PONE-D-24-37086
“Post-Pleistocene Colonisation Rather than the Contemporary Environment has Most
Influenced the Current Population Structure of Scottish Atlantic Salmon”

I have read the manuscript thoroughly; the authors are trying to explain the impacts of deglaciation compared to contemporary environmental changes using Cauwelier et al. (2018) microsatellite data. I also read the previous revisions by 2 different reviewers and the comprehensive responses of authors to the comments. I do not know the background of two previous reviewers but from my point of view, the subject is interesting and the methodology used is brilliant and correct based on the available data. The results and corresponding discussion also seem logical to me. The structure of the manuscript is strong enough after going through two revisions. Finally, I believe the manuscript is full-fits enough and can be published as it is.

We thank this reviewer for their time and for their kind words regarding our manuscript.

Reviewer #4: In this manuscript, the authors attempt to show the relative contributions of historical and contemporary factors affecting genetic diversity and differentiation of Atlantic salmon in Scotland using microsatellite genotypes from populations previously analyzed in Cauwelier et al. (2018). Previous reviewers had called into question some of the methodology used in this manuscript, but I largely agreed with the authors in response to most of these concerns, outlined specifically below. I do, however, think that while the GLMs and GESTE models are valid methods of analyzing this data, the inferences that can be drawn from them are relatively weak, and there are additional methods and alternative approaches that could be used to supplement the authors’ findings and make the takeaways of the manuscript more convincing. Additionally, I think the writing style lacks specificity in key areas of the introduction, methods and discussion, and as a result parts of the manuscript are difficult to interpret. Overall, this manuscript focuses on clear and relevant ecological questions using an expansive microsatellite dataset, and I would recommend this manuscript for publication with a few minor improvements.

We thank this reviewer for the time they have put into reading our manuscript and the detailed comments they have made, which have prompted us to improve our manuscript greatly.

Major Issues
While I do not share the second reviewer’s concerns about the lack of variability in the environmental data, I do think that the results of the paper are somewhat difficult to interpret because how the environmental variation is spread across the sampled populations is not well described. Table S1 should be moved into the main text of the paper and the authors could consider additional map figures showing the most relevant environmental information for each of the sampled populations.

Table S1 is now Table 1 in the main body of the manuscript.

I agree with the authors that the use of population-specific Fst is absolutely appropriate for these analyses. While I could see some utility of comparing isolation by distance tests with tests of isolation by resistance/isolation by environment using pairwise Fst and/or other genetic distance metrics, because of the coarseness of many of the environmental variables used, I do not think that those tests would be very informative, at least for the goals of this manuscript.

There are other approaches that could be included to strengthen the inferences drawn in the manuscript. Redundancy analysis would be a relatively simple addition to assess the relative importance of environmental factors on genetic variation that could be directly compared to the results from the GLMs and GESTE, as well as show if there are any markers and populations are driving patterns associated with each environmental variable. Coalescence and demographic reconstruction analyses could also be relevant inclusions that could corroborate the timing of glacial retreats being the major factor structuring salmon populations in the study area.

One of the main conclusions of the paper is that contemporary habitat modifications are affecting genetic connectivity of these populations, but the effect size detected by GESTE is small, and analysis of genetic differentiation alone is not likely to demonstrate a strong signal of fragmentation due to relatively recent habitat modifications. This claim in particular would benefit from an analysis comparing patterns in historical gene flow (e.g. using a coalescence-based modelling approach like MIGRATE) versus contemporary gene flow (i.e. methods based on identifying migrants and their offspring in the sampled individuals), and seeing if populations in systems that have had habitat modifications show drastically different historical vs. contemporary patterns of connectivity.

We agree with the reviewer that RDA is potentially useful method to evaluate the relative importance of environmental factors on genetic structure. We have therefore conducted this extra analysis and reported the results in our manuscript. The methods are described on lines 235-239, while the results are reported on lines 314-329. Table 5 includes extra information on the loadings of each explanatory variables and figure 4 serves as a visualisation. We have included discussion of these results (lines 342-345; 364-371), noting that they highlight the fact that, on the east coast, waterbody modification has an effect on the same order of magnitude as time since deglaciation. This is therefore an example of an anthropogenic effect playing an important role in shaping genetic variation on an ecological timescale.

Note, however, that directly comparing MIGRATE migration estimates with those obtained from multilocus approaches based on gametic disequilibrium (e.g., assignment tests, BayesAss or BIMr) is not appropriate as they differ in the type of migration rate they estimate. MIGRATE estimates long-term **forward migration** rates while the gametic disequilibrium methods estimate recent **backward migration** rates. More precisely, MIGRATE estimates

$$M_{ij}=\mu\nu_{ij},$$

where $\mu$ is the substitution rate scaled in units of generations and $\nu_{ij}$ is the fraction of individuals that migrate from population *i* to *j* (Beerli & Felsenstein, 2001).

On the other hand, gametic-disequilibrium methods estimate

$m_{ji}=\frac{N_{i}}{N_{j}}\nu_{ij}$.

Thus, to compare the two types of estimates we need to know the census population sizes of each of the two populations involved in the exchange of individuals, something that is not readily available. It is not uncommon for publications to compare these estimates using ‘guesstimates’ of per generation substitution rates or of *N_e_*, but this practice is not acceptable. In fact, the comparison of the two types of estimates is not a trivial endeavour and would represent a large and laborious departure from the objectives of our study. Thus, we have not implemented this change in order to keep the focus on environmental drivers of migration.

Minor comments:

Abstract

Lines 35-37: Be more specific, what were the effects of waterbody modification? You should also mention what metrics you looked at in the abstract.

Effect of waterbody modifications are now included, along with the list of metrics (lines 34-40).

Introduction

Lines 45-52: This paragraph feels unfocused and does not introduce what this manuscript is about. It might be better to focus on how the environment and demographic processes interact to produce patterns in genetic variation before moving into how these processes tend to manifest in salmon populations specifically. The general biological information about salmonids that affects population genetic processes is important background information, but would be more appropriate for later in the introduction.

We introduce Salmonids into the discussion slightly later but we still introduce them fairly early on as this paper is really about the population genetics of Salmonids (especially *S. salar*) and there is nothing particularly interesting or novel that we have to say about the processes in general. Hopefully what we have now is better (lines 49-63).

Lines 78-100: The previous section of the introduction laying out the natural history of glaciation and the impact on salmonid populations was quite effective at providing context, whereas this section reads more as a point-by-point justification for the environmental variables you included. This section would be more effective if it were reorganized into more of a narrative format like the above section was presented, giving a history of how anthropogenic factors have altered the contemporary landscape affecting these populations.

Done (lines 82-105).

Methods

Lines161-163: It would be good to include a short description of the factors used to classify water quality.

There are dozens of factors so we cannot list them all, but we provide some extra clarity in the manuscript by mentioning the fact that these relate to the chemistry, biology, specific pollutants and hydromorphology of the river in question (lines 169-170). The link in the references will allow readers to easily find out more about the Water Framework Directive if they wish to.

Line163: This brief sentence is the only mention of waterbody modification in the methods. It is very important to describe what how this variable is constructed, especially since it is the most important contemporary effect you discuss later the results and discussion, but readers have no context as to what exactly this variable represents.

This is another fairly complex topic but we have added a sentence explaining that heavily modified waterbodies are permanently physically altered and substantially changed in character due to human activities (lines 172- 175). All sites considered as modified are in our case designated as such under the Water Framework Directive.

Lines 169-171: It is not clear what the difference is between dummy variables 2 and 3. Does 3 include only riparian buffers or any terrestrial area in the watershed? What are the regulations designed to protect salmon versus protective regulations not designed for salmon? Without any details, these variables seem like they could encompass a very wide variety of regulations that would have highly variable effects on salmonid populations and it is hard for readers to assess what the dummy variables are representing.

The difference is that 2 is a designation only for the river itself, which includes the riparian buffer, whereas 3 is for the river and a greater surrounding area. The rationale that the Joint Nature Conservation Committee give for specific protections for salmon is largely based on protecting spawning and nursery requirements for salmon. We have now made this clear in our manuscript (lines 176-182).

Line 177: What is the temporal resolution of this estimate? Is this a temperature that the fish might have experienced for an hour? A day? A month? Choosing the maximum also seems like it would be subject to outlier events that may not reflect the general thermal suitability of a stream. How closely does the max temp co-vary with the average temp?

We have clarified that this is the maximum daily temperature on the hottest year over the last 20 years (lines 192-193). We chose the maximum temperature because we thought that it was the extreme events that would cause events like major fish mortality or inaccessibility to sections of river, as hypothesised in Hand *et al.* (2016).

Lines 177-178: Unclear how temperature is linked to the time since deglaciation.

They are not linked. This is a poorly written sentence on our part and we have now changed it (lines 193-194).

Lines 194: Specify that rarefied allelic richness was the diversity measure used as the dependent variable in the GLMs

Done (line 211).

Lines 204-205: The pilot runs are used as the burn-in period for GESTE. You wouldn’t have a thinning interval for a burn-in period.

Another poorly written sentence on our part which we have now hopefully clarified (lines 221-224).

Lines 219-223: Was there hierarchical structure detected in the Cauwelier et al. 2018 paper? If the east and west coasts represented a major division in your populations that possibly pre-dates deglaciation, that would be good to mention here as justification for splitting up the populations into separate models.

Hierarchical structure was detected but we do not know if this predates deglaciation.

Discussion

Lines 312-355: This section can be substantially reduced and moved to a less prominent place in the discussion. Distance to the sea is not a major factor that appears to be structuring these populations and it would be better to consider models that include the effects of the confounding factors you pointed out (i.e. effective population size) rather than present model results with “outlier” sites removed.

We have reduced the size of this section and moved it to further on in the discussion (lines 383-403).

Lines 339-341: Estimates of effective population sizes would strengthen your inferences about the potential influence of genetic drift in your populations.

Estimates of Ne based on microsatellites from samples obtained at a single point in time have very large confidence intervals (e.g., very frequently, the upper bound of linkage-disequilibrium estimates equals infinity) so they are unlikely to provide much information about strength of genetic drift in our case. Thus, we have not incorporated this analysis.

Line 363: You did not test for interaction effects between your environmental variables.

Fixed (lines 363-364).

Lines 368-370: Are “hydro schemes” dams? I would use a more specific descriptive term here.

Hydro scheme is a broad term that includes dams but also ‘run-of-river schemes’ that utilise the natural flow and elevation drop of a river to generate electricity (they do not have any notable storage and are reliant on river levels to generate energy). It also includes ‘pump storage schemes’ that produces energy by transferring water from an upper reservoir or raised loch to a lower reservoir or loch during periods of high demand. More detailed explanations can be found at <https://www.sepa.org.uk/media/152049/wat-sg-74.pdf>.. We have now also made changes on lines 374-376 so that it is clear to the reader that hydro schemes are not just dams.

Lines 368-376: It seems obvious to me that dams would have very different effects on connectivity compared to irrigation canals and dykes. If you have this information, why not parameterize your models specifically to look at the effects of dams versus other waterbody modifications?

This is a bit tricky. As stated before, hydro schemes are not just dams. The Water Framework Directive designation could also be referring to the abstraction of water which reduces the carrying capacity of the river below a dam and this is likely to have an impact on genetic diversity and differentiation. Arguably this can have far more of an impact than a semi-passable barrier. This will vary however. Furthermore, water is often transported between catchments. Several of our sites are being impacted by hydro schemes, sometimes because of barriers, in other cases because of the changing water levels, and in further cases, because of changes to the riverscape. As can be seen in figure 2 genetic diversity is consistently lower in modified watercourses on the east coast, compared to unmodified waterbodies deglaciated at the same time. We therefore think that, given our current knowledge, sticking to the Water Framework Directive designation of a heavily modified waterbody is our best option.

Lines 378-408: This section can be substantially reduced.

One paragraph has been removed and others have been shortened. The remaining section is on lines 407-423.

Line 410: Rename this section. As presented, it implies that the previous parts of the discussion only applied to the east coast.

Renamed as ‘Considerations for the west coast’ (line 425).

References

Beerli,P. and Felsenstein, J. (2001) Maximum likelihood estimation of a migration matrix and effective population sizes in *n* subpopulations by using a coalescent approach. *Proceedings of the National Academy of Sciences,* 98: 4563-4568.

Cauwelier, E. Verspoor, E. Coulson, M. W. Armstrong, A. Knox, D. Stradmeyer, L. Webster, L. M. I. and Gilbey, J. (2018) Ice sheets and genetics: insights into the phylogeography of Scottish Atlantic salmon, Salmo salar L.. Journal of Biogeography, 45: 1-13.

Hand, B. K. Muhlfeld, C. C. Wade, A. A. Kovach, R. P. Whited, D. C. Narum, S. R. Matala, A. P. Ackerman, M. W. Garner, B. A. Kimball, J. S. Stanford, J. A. and Luikart, G. (2016) Climate variables explain neutral and adaptive variation within salmonid metapopulations: the importance of replication in landscape genetics. Molecular Ecology, 25: 689-705.

Lewontin, R. C. (1966) On the measurement of relative variability. *Systematic Zoology,* 15: 141-142
