# Supplementary material for: Post-pleistocene colonisation rather than the contemporary environment has most influenced the current population structure of Scottish Atlantic salmon (Salmo salar)
Source: PLoS One. 2025 Oct 1;20(10):e0333164. doi: 10.1371/journal.pone.0333164 (PMC12488018; doi:10.1371/journal.pone.0333164)
Supplement: Attachment — Submitted filename: Response_to_Reviewers_auresp_3.docx [file pone.0333164.s010.docx]

Dear Dr. Finn Cowell; Thank you very much for your revision. Please let me inform you that your submission needs a minor revision before being accepted for publication. In particular, (1) Introduction needs in-brief description on anthropogenic factors that affect genetic diversity and structuring of salmonid populations; (2) Dr Waraniak suggested to run a variance partitioning analysis. I would be very much appreciate it if you kindly revise the manuscript according to the comments appended below.

We are grateful for the feedback received on this submission and we have altered our manuscript to comply with the comments. Please, note that in the process of revising the ms, we realised that an input file for calculating allelic richness did not use the proper coding for missing data. We have corrected this mistake and can confirm that the new results did not change the main conclusions of our analysis.

Reviewer #4: The authors have improved the manuscript somewhat, though there are a few areas that I still have some concerns about before recommending this manuscript for publication. The main issue I have currently is that while the authors added a redundancy analysis as I recommended, they perhaps did not understand the purpose of the RDA and implemented it in a way that does not make much sense and does not provide many new insights compared to the other analyses they present. Additionally, I do not think the authors have fully addressed my concerns around specificity and providing enough appropriate details (particularly in the introduction) on the environmental factors that would allow the audience to make clear links between patterns in the genetic data and the environmental variables and assess how the analyses have been interpreted for themselves. More specific suggestions are listed below:

We thank this reviewer for their continued engagement with our manuscript and the useful comments. We hope the changes we have made in accordance with specific suggestions will allay any concerns.

Major issues
Introduction (Lines 82-105): This listing of the anthropogenic factors is too generic and needs to be more explicit in how the authors might expect these factors to affect the genetic diversity and structuring of salmonid populations especially for the more general audience of PlosONE. E.g. in lines 96-99, dams and aquaculture escapees are introduced in the same sentence saying that they may impact genetic structuring, but the expected effects of these are very different, with dams increasing the risk of inbreeding (lower genetic diversity, possibly greater genetic differentiation) and aquaculture/stocking increasing the risk of outbreeding (increasing genetic diversity and possibly reducing genetic differentiation if the same stocking source interbreeds with different wild populations).

We have linked each of the anthropogenic factors to the evolutionary forces that we expect to have an impact on genetic differentiation and diversity (lines 96-101 and 107-109).

Methods (Lines 235-239): Using RDA in this way is not invalid per se, but is not substantially different from the GLMs. In genetic studies, it is more common to use a matrix of raw genotypes as the dependent variables in an RDA rather than a matrix of summary statistics. RDA is really meant to analyze high-dimensional data (e.g. genotypes from many markers), so using it for two summary statistics does not make a lot of sense. The authors may also wish to run a variance partitioning analysis (see Capblanq & Forester 2021: https://doi.org/10.1111/2041-210X.13722) as the most relevant way to present the results of the RDA for the purposes of their paper. This method will allow you to quantify how much inter-population genetic diversity is explained by your contemporary vs. historical factors and how much may be confounded by collinearity between your environmental factors. You will also be able to see if there are certain microsatellites that appear to be affected by specific environmental factors.

We agree with the reviewer that RDA is typically applied to the genotype matrix, but this is only possible when using biallelic SNPs, in which case genotypes can be coded unequivocally as (0, 1, 2) depending on how many copies of the reference alleles an individual has at a given locus. However, we are working with microsatellites with several alleles at each locus and, therefore we cannot use this simple coding to assign values to each genotype. There is no ideal solution for coding microsatellites in a way that makes them amenable to use in an RDA. Thus, we used an approach implemented in hierfstat, which consists in generating a total of 732 dependent variables by converting the microsatellite data into allele frequencies, with one allele (column) removed for each locus. Three RDA models were run: the first had all variables, the second included contemporary variables and controlled for the effect of the historical variables and the third included historical variables and controlled for the contemporary variables. In this instance, distance to the sea and time since deglaciation were the historical variables. As can be seen in table 1, none of these have a statistically significant impact on the differing frequencies of alleles between populations. We do not think these results are particularly interesting and therefore we have decided not to include them in the manuscript.

Table.1: Results of a variance partitioning analysis using RDA.

| Variables | Canonical Variance | R^2^ | p-value | Proportion of Canonical Variance |
| --- | --- | --- | --- | --- |
| All | 140 | 0.191 | 0.504 | 1 |
| Contemporary | 96.7 | 0.132 | 0.223 | 0.691 |
| Historical | 31.6 | 0.043 | 0.422 | 0.226 |

Minor suggestions

Line 57: replace “and” before Norway with a comma

Done.

Line 123-124: reference Figure 1 here

Done.

Lines 145-149: Figure legend should reference what the colors are depicting

Done.

Line 146: “Table S1” should now be “Table 1”

Done.

Line 215: Were there clear best models (∆AIC > 2 ) for each of the sets of GLMs tested? Also, considering that there are only 48 sites included, AICc for small sample sizes might be more appropriate in this case.

The range of AIC values is > 2 but the difference between the lowest AIC value and the next lowest is < 2. We switched to using AICc, as suggested, and the result of this is that the simplest model was chosen in each case. For all sites and the west coast this resulted in the time since deglaciation being left in the model. For the east coast, waterbody modification was chosen (see table 2 and figure 2).

Lines 322-323: You can calculate the significance of individual explanatory variables in an RDA by running the “anova.cca” function with the ‘by = “terms”’ option.

We thank the reviewer for pointing this out as an option open to us, but we are happy with only presenting the significance between matrices Y and X and then presenting the loadings of the variables for each canonical axis, with the significance of the axes shown in figure 4. It can clearly be seen in table 5 that waterbody modification and time since deglaciation have far greater loadings than any of the other variables. Therefore, we think that all the appropriate information is already in the paper and no more detail is required. We would also like to point out that only time since deglaciation and waterbody modification have significant p-values so the results we are presenting are in no way misleading.
