# Supplementary material for: Post-pleistocene colonisation rather than the contemporary environment has most influenced the current population structure of Scottish Atlantic salmon (Salmo salar)
Source: PLoS One. 2025 Oct 1;20(10):e0333164. doi: 10.1371/journal.pone.0333164 (PMC12488018; doi:10.1371/journal.pone.0333164)
Supplement: Attachment — Submitted filename: Response to PLoS ONE.docx [file pone.0333164.s011.docx]

We are once again very grateful that this reviewer continues to provide us with helpful comments that have allowed us to improve our manuscript.

Reviewer #4: The authors have addressed most of my concerns from the previous round of reviews. I only have two small comments:
1) While it is possible to use multi-allelic loci in an RDA with each allele coded as 0/1/2 (so long as the organism is a diploid; this refers to the count of a specific allele in a genotype, regardless of whether a loci only has 2 possible alleles like a biallelic SNP or more like a multi-allelic microsat), conducting a population-wise RDA using allele frequencies is an equally valid approach that would lead to similar conclusions since the environmental variables were measured at the population level. While the authors may choose not to report the results of that analysis, as there were no significant relationships found between allele frequency patterns and the environmental variables tested, I would consider that result somewhat interesting as a suggestion that isolation by environment has not been a strong force shaping the population structure of these populations. Additionally, the RDA on allelic richness and Fst that was included does not make much sense in this context and I would recommend removing it from the manuscript.

We thank the reviewer for giving us the option on whether or not to include the results of this analysis and we note the point this reviewer makes. However, fundamentally the results from the allele frequency RDA does not change the conclusions that can be drawn from the other analyses already included in the manuscript so we do not include it in this latest version. We do however take the advice of this reviewer and remove the A_r_ and F_st_ RDA from the manuscript.

2) If you are going to use AIC (or AICc? It was unclear whether the model selection procedure methods were updated) as a way to assess models, those results need to be reported at some point in the manuscript. If there was one clear best-performing model in each case, you can minimally report that ∆AIC >2 for all other models tested.

We take this opportunity to clarify that we are using AICc scores (line 220). We have now also included a sentence stating that “The difference between the AICc score of the simplest model and the next lowest score was not > 2 but the simplest model was nonetheless chosen as the best model in each case” (lines 265-266). We hope that this is alright because we would rather not have an entire table dedicated to AICc scores for each model, including model specifications, as this would be very large (5 x 26) and most of the information would be surplus to requirements.
